# Trajectory inference for a branching SDE model of cell differentiation via lineage tracing

**DOI:** 10.1007/s00285-026-02375-5

**Published:** 2026-04-17

**Authors:** Elias Ventre, Aden Forrow, Nitya Gadhiwala, Parijat Chakraborty, Omer Angel, Geoffrey Schiebinger

**Affiliations:** 1 The University of British Columbia, Vancouver, BC Canada; 2 The University of Maine, Orono, Maine USA

**Keywords:** Trajectory inference, Branching processes, Single-cell data analysis, Lineage tracing, Generation numbers, The Schrödinger problem, 60J85, 70F17, 92-10

## Abstract

A core challenge for modern biology is how to infer the trajectories of individual cells from population-level time courses of high-dimensional gene expression data. Birth and death of cells present a particular difficulty: existing trajectory inference methods cannot distinguish variability in net proliferation from cell differentiation dynamics, and hence require accurate prior knowledge of the proliferation rate. Building on Global Waddington-OT (gWOT), which performs trajectory inference with rigorous theoretical guarantees when birth and death can be neglected, we show how to use lineage trees available with recently developed CRISPR-based measurement technologies to disentangle proliferation and differentiation. In particular, when there is neither death nor subsampling of cells, we show that we extend gWOT to the case with proliferation with similar theoretical guarantees and computational cost, without requiring any prior information. In the case of death and/or subsampling, our method introduces a bias, that we describe explicitly and argue to be inherent to these lineage tracing data. We demonstrate in both cases the ability of this method to reliably reconstruct the landscape of a branching SDE from time-courses of simulated datasets with lineage tracing, outperforming even a benchmark using the experimentally unavailable true branching rates.

## Introduction

Over the last twenty years, single-cell RNA-sequencing (scRNA-seq) technologies have opened new windows on the study of cell-differentiation processes by offering the possibility of measuring, for a population of cells, the numbers of mRNA molecules expressed by the genes of each individual cell. In probabilistic terms, these *single-cell data* give access to a joint probability distribution of gene expression, instead of the average value for each gene provided by population-level data (Mar 2019; Coskun et al. 2016). A major limitation is that the observation process is destructive: instead of observing the trajectory of cells directly, we can only observe the state, in the so-called gene expression space, of independent cells at different timepoints. Single-cell data can then be seen as temporal marginal distributions of a stochastic process characterizing the differentiation process. Trajectory inference is the challenging problem of recovering the full dynamic process from the limited static snapshots.

A large and rapidly growing set of methods have been proposed for inferring trajectories using a wide range of computational tools, including diffusion maps (Haghverdi et al. 2016), recurrent neural networks (Hashimoto et al. 2016), and kernel regression (Qiu et al. 2022). The algorithms are commonly based on heuristic biological intuition and rarely come with rigorous theoretical guarantees. One exception is the optimal transport based theory introduced with Waddington-OT (WOT) (Schiebinger et al. 2019), which reconstructs the associated time-varying probabilistic distribution from time-courses of potentially sparse high-dimensional gene expression data. Lavenant et al. (2021) provided a careful mathematical foundation for a variant of WOT named global Waddington-OT (gWOT). In particular, under the hypothesis that the differentiation process can be modeled by a Stochastic Differential Equation (SDE) where the drift has zero curl (and hence is the gradient of some potential as in equation (1) below), they proved that the reconstructed measure converges to the ground truth as the number of timepoints at which the cells are measured goes to infinity.

However, the analysis by Lavenant et al does not account for cellular proliferation and death. Extending the theory to the case of *branching processes* is a major challenge when the proliferation and death of cells allow for variations in mass that are nonuniform across the gene expression space. In the theory of gWOT, the authors presented a way to adjust for proliferation if the ground truth growth rates were known before the experiment (Lavenant et al. 2021; Zhang et al. 2022), but their method presented two major drawbacks: i) it was very sensitive to the prespecified proliferation rate and ii) convergence to the ground-truth path-measure was no longer guaranteed. In biological systems, the characteristics of the SDE and the branching rates are strongly intertwined, which makes disentangling them particularly challenging for inference from sc-RNAseq data alone that does not provide specific information about proliferation and death. To the best of our knowledge, all existing methods that attempt to account for branching require prior knowledge of the branching rates (Weinreb et al. 2018; Zhang et al. 2022).

In this paper, we show how recent *lineage tracing* technologies, which measure not only cell state but also the lineage relationships in a population of cells (Mckenna et al. 2016; Raj et al. 2018) provide a solution to the growth rate problem in trajectory inference. One approach to lineage tracing leverages CRISPR-Cas9 to continuously mutate an array of synthetic DNA barcodes that have been incorporated into the chromosomes so that they are inherited by daughter cells. By analyzing the pattern of mutations in the barcodes of each cell in a population, biologists can reconstruct a lineage tree which describes shared ancestry within the population. At a given time of measurement $t_i$, this lineage tree ${\mathcal {T}}_{t_i}$ is a rooted tree whose leaves correspond to observed cells at $t_i$, each leaf being labeled by its gene expression state. The root corresponds to the ancestor cell at time 0, and internal nodes represent division events. If different ancestor cells are not connected between each other, the observation at $t_i$ becomes a collection of independent lineage trees $\{T_{i}^{(1)}, \ldots , T_{i}^{(K_i)}\}$ ($K_i$ being the number of independent lineage trees at $t_i$). However, it does not contain any direct information about the state of the ancestor cells. Thus, this technology makes accessible a new kind of data, for which the measurement of a population of cells can be seen as a set of *trees* rather than a set of *cells*.

The main result of this article is that this additional lineage information can be used to extend the theory of trajectory inference to branching processes. In the particular case where the cells cannot die and all the leaves of each tree are well observed, we develop a method which is asymptotically exact (in the limit of an infinite number of timepoints at which the cells are collected, as in Lavenant et al. (2021)). We also show that this method allows for an efficient estimation of the characteristics of the branching process, that is the drift of the underlying SDE and the division rates. Importantly, we empirically demonstrate that our method even outperforms the result provided by the gWOT method with full prior knowledge of the branching rates (Zhang et al. 2022). In the general setting where the death rate is nonzero or not all cells are observed, a bias arises in the reconstructed path-measure due to the incompleteness of the information used in the lineage tree. We characterize this bias explicitly and propose a heuristic method to reduce it. Although removing the bias seems completely out of reach with this type of data, we argue and illustrate numerically that this bias is likely to be small, even without applying the heuristic correction.

We emphasize that these results are very different from the few works previously published in this nascent field of single-cell data analysis with lineage tracing, including LineageOT (Forrow and Schiebinger 2021), COSPAR (Wang et al. 2022) and MOSLIN (Lange et al. 2023). The outputs of all three are different from ours: they use the lineage information to reconstruct couplings between observed cells, while we aim to use this information to reconstruct the path-measure of a stochastic process modeling cell differentiation, and the couplings only appear as one part of this reconstructed path-measure. Critically, like all other trajectory inference methods we are aware of, they cannot disentangle variability in proliferation rates from biases in differentiation.

### Mathematical overview

In this article, we model the biological process of cellular differentiation, in a time interval [0, *T*], with a branching stochastic differential equation (branching SDE).

More precisely, each cell is described at any time $t \in [0,T]$ by a random vector $X_t$ specifying its cell state. This vector belongs to the so-called gene expression space ${\mathcal {X}} \subset {\mathbb {R}}^g$, where *g* is the number of genes of interest, and follows the law of an SDE. We assume that the drift of the SDE is the gradient of some potential $\Psi$ defined from $[0,T] \times {\mathcal {X}}$ to ${\mathbb {R}}$, and that the diffusion coefficient is a constant $\tau$. The spatial evolution of the cell state $X_t \in {\mathcal {X}}$ follows

1 $$\begin{aligned} \mathrm d X_t = -\nabla \Psi (t, X_t)\mathrm dt + \sqrt{\tau } \mathrm d B_t. \end{aligned}$$

We denote by $P^\tau \in {\mathcal {P}}({\mathcal {C}}([0, T], {\mathcal {X}}))$ the path-measure characterizing this SDE.

Moreover, to take into account proliferation, cells can divide into two independently evolving cells or die at exponential branching rates *b*(*t*, *x*) and *d*(*t*, *x*) respectively. The branching SDE is then measure-valued (see Section 2 for more details), and the path-measure characterizing it belongs to ${\mathcal {P}}(\text {cdlg}([0, T], {\mathcal {M}}_+({\mathcal {X}})))$, where ${\mathcal {M}}_+({\mathcal {X}})$ denotes the space of positive measures on ${\mathcal {X}}$.

The problem of trajectory inference consists in reconstructing this path-measure from a sequence of temporal marginal distributions of the process at *N* times $0\le t_1<\cdots <t_N\le T$, which we denote $\mu :=(\mu _{t_1},\cdots ,\mu _{t_n})$. The temporal marginal distributions are positive measures on ${\mathcal {X}}$, which do not necessarily sum to one due to branching and death. In our setting, they correspond to the experimental observations performed by biologists.

The methods WOT (Schiebinger et al. 2019) and gWOT (Lavenant et al. 2021) that we build on in this article both address the case where there is neither branching nor death and the path-measure is simply $P^\tau$. WOT aims to reconstruct $P^\tau$ by minimizing the *relative entropy*, defined by

2 $$\begin{aligned} H(R|W^\tau ) := {\left\{ \begin{array}{ll} {\textbf{E}}^{W^\tau }\left[ h\left( \frac{\mathrm dR}{\mathrm dW^{\tau }}\right) \right] & \text { if }R \ll W^\tau ,\\ +\infty & \text { otherwise,} \end{array}\right. } \end{aligned}$$

among all the path-measures $R\in {\mathcal {P}}({\mathcal {C}}([0, T], {\mathcal {X}}))$ that match the sequence of prescribed temporal marginal distributions. Here, $W^{\tau }$ is the path-measure of a Brownian motion with diffusivity $\tau$ and ${\textbf{E}}^{W\tau }$ denotes the expected value under the law of $W^{\tau }$. The function *h* is defined by^1^$h:\, x \rightarrow x\ln x + 1 - x$, and $\frac{\mathrm dR}{\mathrm dW^{\tau }}$ is the standard Radon-Nikodym derivative between the two path-measures. This minimization problem is generally known as the *Schrödinger problem*, and has been the object of many studies in the last ten years due to its connections with optimal transport (Gentil et al. 2017; Peyré and Cuturi 2019; Léonard 2014).

In practice however, the temporal marginal distributions can only be approximated by the empirical distributions describing the sets of cells that are observed at each time of measurement rescaled by the number of trees they belong to. We denote ${\hat{\mu }}:= ({\hat{\mu }}_{t_1},\cdots ,{\hat{\mu }}_{t_N})$ the resulting sequence of empirical probabilistic distributions.

The method gWOT improves on WOT by considering the case where the number of cells characterizing each ${\hat{\mu }}_{t_i}$ is small. Lavenant et al. show that the marginal *constraints* in the Schrödinger problem (2) should be replaced in that case by marginal *penalizations*. In particular, one of their main results (Theorem 2.3 of Lavenant et al. 2021) is that $P^\tau$ coincides with the minimum of the functional

3 $$\begin{aligned} F_{N,\lambda ,h}({\hat{\mu }}):\, R\rightarrow \tau H(R|W^{\tau }) + \frac{1}{\lambda }\sum \limits _{i=1}^N ({t_{i+1} - {t_i}})H(\Phi _h * {\hat{\mu }}_{t_i}\big | R_{t_i}), \end{aligned}$$

in the limit $N \rightarrow \infty$, followed by $\lambda \rightarrow 0, h \rightarrow 0$. Here, $\Phi _h$ denotes a Gaussian kernel of width *h*, $*$ stands for the convolution product, and $R_{t_i}$ are the temporal marginals of the path-measure *R* at time $t_i$. This result can be seen as a *consistency result* of the gWOT method, which consists in minimizing the functional (3) given a sequence of experimental observations.

When we take into account cell proliferation and death, it is still possible to define the relative entropy by replacing the Brownian motion by a branching Brownian motion (BBM). The associated *unbalanced Schrödinger problem* has been studied in depth recently (Baradat and Lavenant 2021), but there remain important obstacles to applying the new results to the theory of trajectory inference. In particular, the algorithm minimizing the functional (3) requires solving the Schrödinger problem, and simple methods for finding a solution when the reference measure is a Brownian motion (like the well-known Sinkhorn algorithm Sinkhorn 1967; Cuturi 2013) seem unusable in the case with branching (see Baradat and Lavenant 2021, Section 6). Moreover, the drift associated to the resulting optimal path-measure depends explicitly on the choice of the branching rates of the reference BBM (*ibid*, Section 4), which prevents us from having a consistency result similar to Theorem 2.3 of (Lavenant et al. 2021). These limitations highlight the need for additional information to extend the theory of trajectory inference to the branching case.

We show in this article that the information provided by lineage tracing suffices to account for branching, at least in some particular cases. One of our main results is Theorem 7, which is an extension of Lavenant et al. (2021), Theorem 2.3, and can be summarized as follows:

#### Theorem 1

With the previous notation, if the death rate $d = 0$, we can build, from the sequence of empirical (non-probabilistic) distributions $({\hat{\mu }}_{t_i})_{i=1\cdots ,N}$ obtained from independent copies of the branching process sampled at different times and the corresponding sequence of collections of lineage trees, a sequence of empirical probabilistic distributions ${\hat{p}} = ({\hat{p}}_{t_i})_{i=1\cdots ,N}$ such that the minimizer $R_{N,\lambda ,h}$ of $F_{N,\lambda ,h}({\hat{p}})$ converges narrowly to $P^{\tau }$ in ${\mathcal {P}}({\mathcal {C}}([0, T], {\mathcal {X}}))$ in the limit $N \rightarrow \infty$, followed by $\lambda \rightarrow 0, h \rightarrow 0$.

A key point of interest in this result is that the convergence does not depend on the number of trees or cells constituting the empirical observations. Its proof relies on a simple, important, and to the best of our knowledge never explicitly pointed out, observation: if we observe a set of trees corresponding to independent realizations of a branching process, knowing the *observable generation number* ${\tilde{m}}(x)$ of each cell *x*, i.e. the number of divisions recorded in a lineage tree that cell *x* underwent before its measurement, enables reconstructing the time-varying distribution of a new stochastic process without proliferation. This process coincides:

1. with the real underlying SDE (1) when there is no death and we observe all the leaves of each observed tree;
2. with a new SDE with a modified drift when the cells can die and/or the observed cells are a subsample of the real number of cells corresponding to each tree.

Case 1 presented above is precisely the case for which Theorem 1 applies, after a slight extension of existing results. For case 2, we describe in Section 5 the bias arising in this situation, which depends on the survival probabilities of the branching process with death and subsampling. Our main finding is that provided that every initial cell at time 0 has at least one observed descendant at every timepoint, the reweighted empirical measures (15) converges, in the limit of an infinite number of trees, to the time-varying distribution of an SDE with a drift bias that follows the system of master equation (27) (see Corollary 11). We also present a numerical method for controlling the bias using the times of last common ancestors for each pair of leaves of the lineage trees.

**Fig. 1:**
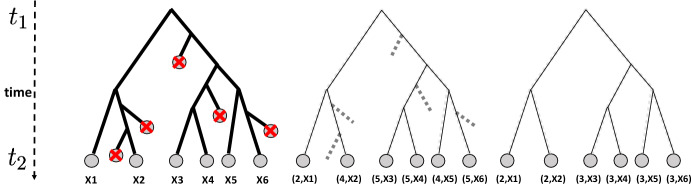
"Different representations of the leaves of a tree evolving between $t_1$ and $t_2$, represented in (A), using: (B) the real generation numbers *m*(*X*) associated to every leaf *X* and (C) the observable generation numbers ${\tilde{m}}(X)$ associated to every leaf *X*. Note that if no cell dies between $t_1$ and $t_2$, the representations in (B) and (C) coincide.

### Organization of the paper

In Section 2, we describe the experiments providing single-cell data with lineage tracing, and detail the subsequent mathematical assumptions. In Section 3, we present the main idea of this article, that is to use generation numbers to *deconvolve* the proliferation, *i.e.* to find the distribution of the underlying stochastic process with only diffusing and vanishing cells. In Section 4 we present the theoretical guarantees associated to the case of null death rate and no subsampling. We illustrate how our deconvolution method compares with the heuristic method for trajectory inference with branching presented in Zhang et al. (2022), which required the knowledge of the branching rates instead of lineage tracing, and show that our results also allow for an efficient numerical reconstruction of the drift and the birth rate characterizing the branching SDE. Section 5 is devoted to the analysis of the bias which appears in case of death and subsampling, and how to reduce it.

## Experimental setting and mathematical assumptions

It has long been impossible in cell biology to generate experimental datasets such that both the gene expression at the single-cell level and information about lineage relationships are available. But these last few years, measurement technologies have seen tremendous recent advances, and it is now possible to recover the full lineage tree of a population of cells (Mckenna et al. 2016; Raj et al. 2018).

### Description of the experimental setting

Technologies for reconstructing cellular lineage trees use CRISPR–Cas9 genome editing technology to continuously mutate an array of synthetic DNA barcodes. These barcodes are incorporated into the chromosomes so that they are inherited by daughter cells. They are then further mutated over the course of development, in such a way that when a population of cells is measured with RNA-sequencing, analyzing the pattern of mutations in the barcodes of each cell allows reconstructing a lineage tree which describes shared ancestry within the population. Moreover, as DNA barcodes are expressed as transcripts, they can be simply recovered together with the rest of the transcriptome with scRNA-seq.

As is usually the case with RNA-sequencing, cells must be lysed before information about their state or lineage is recovered. Thus, this measurement technology is destructive: the data at each timepoint are independent in that a cell observed at a given timepoint can only share ancestors with cells observed at the same timepoint.

The data is therefore a sequence of independent arrays (one for each timepoint). At each timepoint $t_i$, the corresponding array is of size $N_i \times g$ where $N_i$ is the number of cells observed, perhaps of order $1e3-1e4$, and *g* the number of genes observed, typically $\sim 1e4$. Each coordinate of this array corresponds to the number of “reads" of mRNAs that are measured.

Moreover, the barcodes attached to the reads which enable identification of the cells in classical RNA-sequencing technology here also enable reconstruction of the lineage tree of shared ancestry. The problem of reconstructing the lineage trees from these mutated barcodes is itself a challenge, for which recent tools have shown high efficiency (Chan et al. 2019; Weinreb and Klein 2020), including when the number of cells is very high (Konno et al. 2022). We assume in this paper that we have access at each timepoint $t_i$ to a lineage tree ${\mathcal {T}}_{t_i}$, and we focus on using this information for trajectory inference.

Importantly, although at each measured timepoint we have a tree associated to a population of cells, the information provided about both this tree and the positions of its leaves in the gene expression space is not complete. First, only a fraction of the reads expressed by a cell are sampled, which induces noise in the observation of the cellular states. Second, divisions may happen more often than barcodes mutate, generating some errors in the tree reconstruction. Third, because only a small fraction of the descendants of the cells initially present in the experiment are sampled at each timepoint, we observe only a subset of the nodes in the true lineage tree. The two first problems are likely to generate some noise in the data, the analysis of which is out of the scope of this article. The first one in particular is a classical problem in scRNA-seq data for which standards preprocessing methods have been developed (Butler et al. 2018), and we expect the second one to be considered in the same way. The third problem, called *subsampling* in the following, is specific to lineage tracing technologies and, to the best of our knowledge, its implications have never been properly studied. We will show that it induces a bias in the trajectory inference that we will study carefully in Section 5.

### Description of the mathematical setting

We consider that the time-course of gene expression profiles, which we observe together with their lineage trees at an increasing sequence of timepoints $\{t_1, \cdots , t_N\}$, corresponds to independent realizations of a branching SDE with values on the gene expression space ${\mathcal {X}} \subseteq {\mathbb {R}}^g$, on which we have subsampled a certain number of cells. We denote the last time of measurement by $T:=t_N$.

Ignoring for the moment the lineage tree and considering only the leaves of each tree, the data can be described by the superposition of two processes: first, a measure-valued process of law $P \in {\mathcal {P}}(\text {cdlg}([0, T], {\mathcal {M}}_+({\mathcal {X}})))$, corresponding to the branching SDE; and second, a subsampling process on the realizations of the branching SDE at the observed timepoints. We will describe this model first, including how the subsampling is taken into account. In the next section we will detail how the lineage tree is taken into account through the generation numbers.

#### Description of the model

The branching SDE process we consider has two main characteristics:

- **The motion**: During their lifetime, each cell moves around in ${\mathcal {X}}$, independently of the other cells, following a SDE of the form (1);
- **The branching mechanism**: Each cell has an lifetime which is independent from the other cells, and is exponentially distributed: given that a cell is alive in *x* at time *t*, it divides into two cells at time $t + \delta t$ with probability $b(t, x)\delta t + o(\delta t)$, and dies with probability $d(t, x)\delta t + o(\delta t)$. When it divides at *x*, it gives rise to two cells at *x* that evolve independently.

As a consequence, a branching SDE satisfies these two properties, that are key for its analysis:

- **The Markov property**: Let $({\mathcal {F}}_t)_{t \in [0,T]}$ be the natural filtration associated to the process, let $t \in [0,T]$, and let $t^*$ be a stopping time with respect to this filtration such that $t^* \le t$ *P*-a.s. For any measurable function *F* from ${\mathcal {M}}_+({\mathcal {X}})$ to ${\mathbb {R}}^+ \cup \{+\infty \}$, we have:
  4 $$\begin{aligned} {\textbf{E}}^P\left[ F(Z_t)\big | {\mathcal {F}}_{t^*}\right] = {\textbf{E}}^P_{Z_{t^*}}\left[ F(Z_{t - t^*})\right] , \end{aligned}$$
  where $Z_t = \sum \delta _x$ denotes the random measure formed by Dirac masses at the positions of the cells at time *t*. ${\textbf{E}}^P_{Z_{t^*}}\left[ \cdot \right]$ denotes the expected value, under the law of *P*, starting from the initial condition $Z_{t^*}$.
- **The branching property**: Let $t \in [0,T]$, and denote ${\mathcal {N}}(t) = \text {Supp}\,Z_t$. For any sum of Dirac masses $\mu$ in ${\mathcal {X}}$ and any measurable function *u* from ${\mathcal {X}}$ to ${\mathbb {R}}^+ \cup \{+\infty \}$, we have:
  5 $$\begin{aligned} {\textbf{E}}^P\left[ \prod \limits _{X \in {\mathcal {N}}(t)}u(X)\big | Z_0 = \mu \right] = \prod \limits _{x \in \text {Supp}\,\mu } {\textbf{E}}^P\left[ \prod \limits _{X \in {\mathcal {N}}(t)}u(X)\big | Z_0 = \delta _x\right] . \end{aligned}$$

We define a notion of *distribution* for such processes as follows:

##### Definition 2

We define the intensity of the branching SDE at time *t*, ${\textbf{E}}^P[Z_t]$, as the measure defined on the Borel sets $A \subset {\mathcal {X}}$ as: ${\textbf{E}}^P[Z_t]:\, A \rightarrow {\textbf{E}}^P[Z_t(A)]$. The intensity naturally induces a time-varying distribution on ${\mathcal {X}}$, given by the measure ${\textbf{E}}^P[Z_t]$ at each time $t \ge 0$.

Equivalently, under assumption 3, ${\textbf{E}}^P[Z_t]$ is the measure of ${\mathcal {M}}_+({\mathcal {X}})$ defined for any function $\theta \in C^b({\mathcal {X}})$ by

$$\begin{aligned} \langle \theta , {\textbf{E}}^P [Z_t] \rangle = {\textbf{E}}^P\left[ \langle \theta , Z_t\rangle \right] , \end{aligned}$$

where $\langle \cdot , \cdot \rangle$ stands for the duality bracket between continuous functions and measures of finite total variation in ${\mathcal {X}}$.

Strictly speaking, throughout the paper we should give cells labels to refer to them, because many cells could be in the same position at the same time. However, when the number of initial cells is finite, Assumption 3 implies that almost surely each cell at one time has a unique state. For the sake of simplicity, we therefore identify each cell at a given time by its position in ${\mathcal {X}}$.

Throughout our article, we will work under the following very classical assumption that the branching mechanisms *b*, *d* are uniformly bounded,

##### Assumption 3

$$||b + d||_{\infty } < \infty $$,

which ensures that the number of cells at each timepoint is almost surely finite. We don’t mention this assumption in the following, since it is implicit in all our results.

As we are going to prove relations between the time-varying distribution of a branching SDE and the time-varying probabilistic distribution of its underlying SDE, it will also be simpler to consider that the initial measure characterizing the process is probabilistic, i.e. has total mass 1:

##### Assumption 4

The random variable $Z_0$ is sampled under a probability law:

$$\begin{aligned} {\textbf{E}}^P[Z_0] = \mu _0 \in {\mathcal {P}}({\mathcal {X}}). \end{aligned}$$

This is in line with the process we observe since life starts with the formation of a single egg. However, we emphasize that our results can be easily extended to the case where $\mu _0({\mathcal {X}}) \ge 1$ by multiplying temporal marginals of the reconstructed path-measures by $\mu _0({\mathcal {X}})$.

We denote in the following ${\textbf{E}}^P_x[\cdot ]$ the expected value under the law of *P* conditionally to $Z_0 = \delta _x$, and ${\textbf{E}}^P[\cdot ]$ when the initial condition is the probabilistic distribution $\mu _0$ given by Assumption 4. When there is no confusion on the law on which we consider the expected values, we omit the *P* in the expected value.

#### Effect of subsampling

We consider that the subsampling at a time $t \in [0,T]$ consists in taking each cell in ${\mathcal {N}}(t)$ with a probability *q*(*t*), constant in ${\mathcal {X}}$. Importantly, we assume that this probability does not depend on the position of the cells in ${\mathcal {X}}$, nor on the number of cells in ${\mathcal {N}}(t)$. We do allow this probability to depend on time, to take into account the fact that we expect a higher proportion of the cells to be subsampled at early times, when the number of cells is low, than later when the true number of cells is expected to be very high. Thus, the subsampling is just an additional layer: denoting by ${\tilde{P}}$ the modified path-measure characterizing the process described by *P* with subsampling, for any Borel sets $A \subset {\mathcal {X}}$ we have:

6 $$\begin{aligned} \textbf{E}^{{\tilde{P}}}[Z_t](A) = q(t) {\textbf{E}}^{P}[Z_t](A). \end{aligned}$$

#### Inverse problem with lineage tracing

With this model in hand, we can rigorously state the problem of trajectory inference from scRNA-seq data with lineage tracing. It is the question of reconstructing the path-measure *P* from a sequence of experimental measures ${\hat{\mu }}:=\{{\hat{\mu }}_{t_i}\}_{i=1\cdots ,N}$ that are assumed to be sampled under the temporal marginals of ${\tilde{P}}$, together with a sequence of collection of lineage trees $\{\mathcal {T}_{t_i}^1,\cdots ,\mathcal {T}_{t_i}^{K_i}\}_{i=1,\cdots ,N}$ describing at each timepoint the shared ancestry of the cells constituting the associated empirical measure.

The first and main challenge arising from this inverse problem is how to use the lineage tree information to formulate a minimization problem that could characterize the path measure *P* in the limit $N \rightarrow \infty$. As mentioned in the introduction, we show in this article that the generation numbers from the lineage tree suffice for stating and solving a minimization problem characterizing not directly *P* but the path-measure of the underlying SDE, $P^\tau$, at least under some conditions.

We are now going to detail how to take into account these numbers in a dynamical way, by integrating them to the description of the branching process.

## Deconvolving the proliferation using generation numbers

In this section, we extend the description of a branching SDE presented in Section 2 to the case where generation numbers are observed. To make the mathematical presentation cleaner, we start by considering the real generation numbers (Fig. 1.B), and postpone discussion of the partial generation numbers coming from lineage tracing (Fig. 1.C) to Sect. 5. The two types of generation numbers coincide only for experiments without subsampling or death.

The main result of this section could be generalized to any branching process with only birth and death mechanisms, not only branching SDEs.

### Mathematical description and master equation of a branching SDE with real generation numbers

For the branching SDE with real generation numbers, the SDE (1) still characterizes the motion, and the rates *b*, *d* of the branching mechanism. The only difference is that a counter is attached to each leaf of the tree recording the number of divisions the cell has been through. For any time $t \in [0,T]$, ${\mathcal {N}}(t)$ now denotes a set of cells each described by a tuple (*m*, *x*) containing the generation number $m\in {\mathbb {N}}$ and position $x\in {\mathcal {X}}$. The process can thus be described by a collection of random variables $Z_t:= \{Z_t(m)\}_{m\in {\mathbb {N}}}$ each describing the empirical measure on ${\mathcal {X}}$ of the process for a given generation number *m*:

7 $$\begin{aligned} Z_t(m) = \sum \limits _{(m, x) \in {\mathcal {N}}(t)}\delta _{x}. \end{aligned}$$

A realization of a branching SDE is then a ${\mathcal {C}}$ curve valued in ${\mathcal {M}}_+({\mathcal {X}})^{{\mathbb {N}}}$, the jumps of which correspond to the branching events.

The time-varying distribution of the branching SDE with real generation numbers is defined as a natural extension of (2). Let *P* be the path-measure of the branching process with real generation numbers. For any $m \in {\mathbb {N}}$, the intensity at time *t*, written ${\textbf{E}}^P[Z_t(m)]$, is the measure on ${\mathcal {X}}$ defined by

8 $$\begin{aligned} \langle \theta , {\textbf{E}}^P [Z_t(m)] \rangle = {\textbf{E}}^P\left[ \langle \theta , Z_t(m)\rangle \right] \end{aligned}$$

for any test function $\theta \in C({\mathcal {X}})$. This intensity naturally induces a time-varying collection of distribution on ${\mathcal {X}}$, given by the collection of measures $\{{\textbf{E}}^P[Z_t(m)]\}_{m \in {\mathbb {N}}}$ at each time $t \ge 0$.

The initial condition of Assumption 4 then becomes:

$$\begin{aligned} {\left\{ \begin{array}{ll} {\textbf{E}}^P[Z_0(m)] = \mu _0 & \text {if }m = 0,\\ {\textbf{E}}^P[Z_0(m)] = 0 & \text {otherwise}. \end{array}\right. } \end{aligned}$$

Our next goal is to derive a system of master equations characterizing the collection of distributions $({\textbf{E}}^P[Z_t(m)])_{m \in {\mathbb {N}}}$. We start from the classical result that if the potential $\Psi$ has a gradient $\nabla \Psi$ that is globally Lipschitz in ${\mathcal {X}}$, the probability distribution characterizing the SDE (1) solves the following partial differential equation (PDE) in the weak sense:

9 $$\begin{aligned} \partial _t p =&div(p \nabla \Psi ) + \frac{\tau }{2}\Delta p := {\mathcal {L}}^*_{\Psi , \tau } p. \end{aligned}$$

Moreover, the time-varying distribution of the branching SDE described in Section 2 solves the following PDE in the weak sense:

10 $$\begin{aligned} \partial _t \rho = {\mathcal {L}}^*_{\Psi , \tau } \rho + (b - d)\rho . \end{aligned}$$

A proof of this claim can be found for example in Baradat and Lavenant (2021) (Corollary 2.39).

For the branching SDE with real generation numbers, we prove the following proposition:

#### Proposition 5

The collection of distributions $\{{\textbf{E}}^P[Z_t(m)]\}_{m \in {\mathbb {N}}}$ solves the following system of PDEs in the weak sense:

11 $$\begin{aligned} \forall m \ge 0:\, \partial _t \rho (m) = {\mathcal {L}}^*_{\Psi , \tau }\rho (m) + 2b \rho (m-1) - (b + d)\rho (m), \end{aligned}$$

with the convention $\rho (-1, \cdot ) = 0$, and initial condition $\rho _0(m) = \mu _01\!\!1_{m=0}$.

Before detailing the proof, we remark that as expected, the sum $\sum \limits _{m \ge 0} \rho (m)$ solves Eq. (10). Moreover, this equation can be understood as follows:

- At any time, the motion characterizing the branching SDE with real generation number is the same as for the branching SDE;
- When the exponential clock of a cell in (*m*, *x*) rings, it necessarily dies or gives rise to two cells in $(m+1, x)$.

We only give the main elements of the proof, since the details are similar to the ones that can be found in other more complete references, including (Baradat and Lavenant 2021; Li 2011) and Etheridge (2000). We nevertheless give all the main steps that will allow us to extend the result to conditional processes in the next section. The proof relies mainly on the Markov and the branching properties ((4) and (5)).

#### Proof of Proposition 5

The aim is to prove that for any collection of test functions in ${\mathcal {X}}$, $\{\theta (m)\}_{m\in {\mathbb {N}}}$, the collection of distributions $\{{\textbf{E}}^P[Z_t(m)]\}_{m \in {\mathbb {N}}}$ solves the following PDE:

12 $$\begin{aligned} \sum \limits _{m \in {\mathbb {N}}}\frac{\mathrm d}{\mathrm dt}\langle \theta (m), \rho (m) \rangle = \sum \limits _{m \in {\mathbb {N}}} \bigg (&\langle {\mathcal {L}}_{\Psi , \tau } \theta (m), \rho (m) \rangle \nonumber \\&+ \langle \theta (m+1), 2b\rho (m) \rangle - \langle \theta (m), (b+d)\rho (m) \rangle \bigg ), \end{aligned}$$

where ${\mathcal {L}}_{\Psi , \tau }$ is the generator of the underlying SDE (1):

$$\begin{aligned} {\mathcal {L}}_{\Psi , \tau } \theta := -\langle \nabla \Psi , \nabla \theta \rangle + \frac{\tau }{2} \Delta \theta . \end{aligned}$$

It suffices to consider $\theta (m) > 0$, as any $\theta (m)$ could be decomposed into $\theta (m)^+ - \theta (m)^-$ with $\theta (m)^+>0$ and $\theta (m)^->0$.

Let $u_0$ be a function from ${\mathbb {N}} \times {\mathcal {X}}$ to [0, 1] such that for all $m \in {\mathbb {N}}$, $u(m, \cdot )$ is smooth. Let $x \in {\mathcal {X}}$ and $m \in {\mathbb {N}}$. Thanks to the branching property, we can restrict to the case where $\mu _0 = \delta _{(m, x)}$. The proof consists of three steps: (1) find the derivative in $t = 0$ of $u(t, m, x):= {\textbf{E}}_{(m,x)}\left[ \prod \limits _{(M, X) \in {\mathcal {N}}(t)} u_0(M, X)\right]$, (2) deduce that *u* is the classical solution of a certain PDE for any $u_0$, and (3) deduce that the collection of distributions defined by (8) is a weak solution of the PDE (12).

Step 1: Starting from a cell in *x*, at a small time $\delta t$, the probability that the cell has divided is $b \delta t + o(\delta t)$, the probability that that it is dead is $d \delta t + o(\delta t)$, and the probability that no branching event happened is $1 - (b+d) \delta t + o(\delta t)$. Any other event has a probability in $o(\delta t)$. Thus, denoting $A^b, A^d$ and $A^c$ these three complementary events, we have:

$$\begin{aligned} u(\delta t, m, x)&= (1 - (b+d)\delta t) {\textbf{E}}_{(m, x)}\left[ \prod \limits _{(M, X) \in {\mathcal {N}}(\delta t)} u_0(M,X) \big | A^c\right] \\&\quad + b \delta t {\textbf{E}}_{(m, x)}\left[ \prod \limits _{(M, X) \in {\mathcal {N}}(\delta t)} u_0(M,X) \big | A^b\right] \\&\quad + d \delta _t {\textbf{E}}_{(m, x)}\left[ \prod \limits _{(M, X) \in {\mathcal {N}}(\delta t)} u_0(M,X) \big | A^d\right] \\&\quad + o(\delta t),\\&= (1 - (b+d)\delta t) {\textbf{E}}_x^p\left[ u_0(m, X_{\delta t})\right] + b \delta t \left( {\textbf{E}}_x^p\left[ u_0(m+1, X_{\delta t})\right] \right) ^2\\&\quad + d \delta _t + o(\delta t), \end{aligned}$$

where ${\textbf{E}}_x^p$ denotes the expected value under the law of the underlying SDE (1), which is a probabilistic process. The passage from the first to the second line is justified by the branching property. We then obtain:

$$\begin{aligned} \lim \limits _{\delta t \rightarrow 0} \frac{u(\delta t, m, x) - u_0(m, x)}{\delta t}&= \lim \limits _{\delta t \rightarrow 0} \frac{{\textbf{E}}_x^p\left[ u_0(m, X_{\delta t})\right] - u_0(m, x)}{\delta t} \\&- (b+d) u_0(m, x) + b u_0(m+1, x)^2 + d. \end{aligned}$$

The limit on the right-hand side being equal to the generator ${\mathcal {L}}_{\Psi , \tau }$ applied to $u_0(m, x)$, we then obtain the following system of equations:

$$\begin{aligned} \forall m \in {\mathbb {N}}, \forall x \in {\mathcal {X}}:\, \frac{\mathrm d}{\mathrm d t}u(t, m, x)\big |_{t = 0}&= {\mathcal {L}}_{\Psi , \tau } u_0(m,x)\\&\quad \ + b u_0(m+1, x)^2 + d - (b+d) u_0(m, x),\\ &:= {\mathcal {K}}_{\Psi , \tau } [u]_0. \end{aligned}$$

Step 2: Using the Markov and the branching properties of the branching process, we have for all $t, s > 0$:

$$\begin{aligned} u(t+s, m, x)&= {\textbf{E}}_{(m, x)}\left[ {\textbf{E}}_{(m, x)}\left[ \prod \limits _{(M, X) \in {\mathcal {N}}(t+s)} u_0(M,X) \big | {\mathcal {F}}_s\right] \right] , \\ &= {\textbf{E}}_{(m, x)}\left[ {\textbf{E}}_{Z_s}\left[ \prod \limits _{(M, X) \in {\mathcal {N}}(t)} u_0(M,X)\right] \right] , \\ &= {\textbf{E}}_{(m, x)}\left[ \prod \limits _{(M, X) \in {\mathcal {N}}(s)} u(t, M, X)\right] . \end{aligned}$$

Thus, thanks to Step 1, we obtain the following system of PDEs:

13 $$\begin{aligned} \forall m \in {\mathbb {N}}, \forall x \in {\mathcal {X}}:\, \frac{\mathrm d}{\mathrm d s}u(t+s, m, x)\big |_{s = 0} = {\mathcal {K}}_{\Psi , \tau } [u](t, m, x). \end{aligned}$$

Step 3:

Taking $\varepsilon \ll 1$ such that we can define, for all $m \in {\mathbb {N}}$, $u_0(m):= 1 -- \varepsilon \theta (m) \in C({\mathcal {X}}, [0, T])$, we obtain the following relations:

$$\begin{aligned}&u(t, m, x) = 1 - \varepsilon {\textbf{E}}_{(m,x)}\left[ \sum _{m'} \langle \theta (m'), Z_t(m')\rangle \right] + o (\varepsilon ),\\&{\mathcal {L}}_{\Psi , \tau } u(t, m, x) = --\varepsilon {\mathcal {L}}_{\Psi , \tau } {\textbf{E}}_{(m,x)}\left[ \sum _{m'} \langle \theta (m'), Z_t(m')\rangle \right] + o (\varepsilon ),\\&b u(t, m+1, x)^2 + d - (b+d) u(t, m, x) = b + d - (b + d) \\ &\quad -\varepsilon \left( 2b{\textbf{E}}_{(m+1,x)}\left[ \sum _{m'} \langle \theta (m'), Z_t(m')\rangle \right] - (b+d) {\textbf{E}}_{(m,x)}\left[ \sum _{m'} \langle \theta (m'), Z_t(m')\rangle \right] \right) \\&\quad \ + o(\varepsilon ). \end{aligned}$$

Thus, we obtain by substituting in (13) the terms appearing on the left-hand side of the three previous relations by the right-hand side formulas:

$$\begin{aligned} \frac{\mathrm d}{\mathrm dt} {\textbf{E}}_{(m,x)}\left[ \sum _{m'} \langle \theta (m'), Z_t(m')\rangle \right] =&{\mathcal {L}}_{\Psi , \tau } {\textbf{E}}_{(m,x)}\left[ \sum _{m'} \langle \theta (m'), Z_t(m')\rangle \right] \\&+2b{\textbf{E}}_{(m+1,x)}\left[ \sum _{m'} \langle \theta (m'), Z_t(m')\rangle \right] \\ &-(b+d) {\textbf{E}}_{(m,x)}\left[ \sum _{m'} \langle \theta (m'), Z_t(m')\rangle \right] ,\\ :=&\tilde{{\mathcal {K}}}_{\Psi , \tau }\left[ {\textbf{E}}_{(m,x)}\left[ \sum \limits _{m'}\langle \theta (m'), Z_t(m')\rangle \right] \right] . \end{aligned}$$

By the Hille-Yosida theorem, we have then:

$$\begin{aligned} \tilde{{\mathcal {K}}}_{\Psi , \tau } \left[ {\textbf{E}}_{(m,x)}\left[ \sum \limits _{m'}\langle \theta (m'), Z_t(m')\rangle \right] \right] = {\textbf{E}}_{(m,x)}\left[ \sum \limits _{m'}\langle \tilde{{\mathcal {K}}}_{\Psi , \tau }\left[ \theta (m')\right] , Z_t(m')\rangle \right] , \end{aligned}$$

Thus, we can finally conclude that for all initial condition $\delta _{(m, x)}$, the collection of distributions defined by (8) solves the PDE (12). $\square$

### Reconstruction of the temporal marginal distributions of a branching SDE without proliferation

The following corollary of Proposition 5, although very simple, is at the core of our work:

#### Corollary 6

Let the collection of distributions $\{\rho (m)\}_{m\in {\mathbb {N}}}$ be a weak solution of the system (11), and let us denote ${\bar{\rho }} = \sum \limits _{m \ge 0} \frac{1}{2^m} \rho (m)$. Then ${\bar{\rho }}$ solves in the weak sense the following PDE:

14 $$\begin{aligned} \partial _t {\bar{\rho }} = {\mathcal {L}}^*_{\Psi , \tau } {\bar{\rho }} - d{\bar{\rho }}. \end{aligned}$$

#### Proof

The proof of this corollary is a straightforward application of Proposition 5. Indeed, we observe that for all $M > 0$:

$$\begin{aligned} \partial _t \sum \limits _{m =0}^M \frac{1}{2^m} \rho (m) = {\mathcal {L}}^*_{\Psi , \tau } \left( \sum \limits _{m =0}^M \frac{1}{2^m} \rho (m)\right) - d\sum \limits _{m =0}^M \frac{1}{2^m} \rho (m) - \frac{b\rho (M)}{2^M}. \end{aligned}$$

Under Assumption 3, for any $t < T$ and all *m* the mass of the distribution $\rho _t(m, \mathrm dx)$ is finite, and $\frac{b}{2^m}\rho _t(m,\mathrm dx)$ then converges to 0 as $m \rightarrow \infty$. $\square$

In plain words, the observation of generation numbers allows us to deconvolve the proliferation of cells. In particular, under Assumption 4, if the death rate is null, the time-varying distribution $\sum \limits _{m =\ge 0} \frac{1}{2^m} \rho (m)$, which we call the *reweighted distribution* in the remainder of the paper, coincides with the time-varying distribution of the underlying SDE (1). This result allows us to state the theorem of trajectory inference for lineage tracing data.

## Trajectory inference from lineage tracing data with neither death nor subsampling

In this section, we present our analog of the main theorem developed in Lavenant et al. (2021). Theorem 7 below provides guarantees for trajectory inference using single-cell data with lineage tracing in the case where the death rate is uniformly zero and there is no subsampling. Following the proof of the theorem in Sect. 4.1, we then describe how to adapt the mean field Langevin approach of Chizat (2022) into a computationally tractable algorithm for estimating the drift and branching rates of the underlying process. We point out that this second part is heuristic and intends to provide both intuition and numerical evidence rather than formal proofs.

### Consistency theorem

We consider that $K_i$ independent lineage trees at each timepoint $t_i$ are sampled independently from the branching process with $d=0$, and for each tree we observe every leaf. As presented in the introduction, our first and main theorem states that in such circumstances, we can reconstruct the path-measure of the underlying SDE (1) from lineage tracing when the sequence of timepoints tends to be dense in [0, *T*]. When no leaves are unobserved, the real generation numbers and the observable generation numbers *m* and ${\tilde{m}}$ are the same (see Fig. 1); to keep expressions cleaner, we keep the notation *m*.

#### Theorem 7

Let $P^{\tau }$ be the path-measure associated to the SDE (1). and $W^{\tau }$ be the law of a Brownian motion with diffusivity $\tau$. For all timepoints $t_i$, let

15 $$\begin{aligned} {\hat{p}}_{t_i} = \frac{1}{K_i}\sum \limits _{k=1}^{K_i} \left( \sum \limits _{j=1}^{n_{k, i}}\frac{1}{2^{ m_{j,k, i}}}\delta _{x_{j,k,i}}\right) , \end{aligned}$$

where $K_i > 0$ is the number of trees observed at $t_i$ and the collection of tuples $\{m_{j,k, i}, x_{j,k,i}\}_{j=1,\cdots ,n_{k,i}}$ contain the generation number and gene expression for each leaf *j* from the *k*-th tree observed at time $t_i$, all generated by the branching SDE with diffusivity $\tau$, gradient drift $v = -\nabla \Psi$, birth rate *b* and uniformly zero death rate. Let $R_{N,\lambda ,h}$ denote the minimizer of $F_{N,\lambda ,h}({\hat{p}})$ defined by (3).

Then, in the limit $N \rightarrow \infty$, followed by $\lambda \rightarrow 0, h \rightarrow 0$, $R_{N,\lambda ,h}$ converges narrowly to $P^{\tau }$ in ${\mathcal {P}}({\mathcal {C}}([0, T], {\mathcal {X}}))$.

#### Proof

This proposition follows from combining the work of Lavenant et al. (2021) with Corollary 6. By the law of large numbers, the following weak convergence holds: for any continuous function *a* from ${\mathbb {N}} \times {\mathcal {X}}$ to ${\mathbb {R}}$ and the collection of non-negative weights $\{\omega _{i}^N:= t_{i+1} - t_i\}_{i=1,\cdots ,n}$, we have

$$\begin{aligned} \lim _{N \rightarrow \infty } \sum \limits _{i = 1}^N \omega _{i}^N \sum \limits _{m=0}^\infty \int _{{\mathcal {X}}}a(m, x){\hat{\mu }}_{t_i}^h(m, \mathrm dx) = \int _{[0, T]}\sum \limits _{m=0}^\infty \int _{{\mathcal {X}}}a(m,x)\Phi _h * \rho _t(m, \mathrm dx)\mathrm dt, \end{aligned}$$

where ${\hat{\mu }}^h_{t_i}(m, \mathrm dx):= \Phi _h * \frac{1}{K_i} \left( \sum \limits _{k=1}^{K_i} \sum \limits _{j=1}^{N_{k, i}} \delta _{(m_{j,k,i}, x_{j,k,i})}\right)$ denotes the empirical observations, and $\rho$ is the weak solution of the PDE system (11) with initial condition $\mu _0 1\!\!1_{m=0}$.

Thus, for any continuous function *f* from $\mathcal {X}$ to $\mathbb {R}$, we may choose $a(m,x) = \frac{f(x)}{2^m}$ to obtain the following weak convergence:

16 $$\begin{aligned} \lim _{N \rightarrow \infty } \sum \limits _i^{N} \omega _i^N \int _{{\mathcal {X}}}f(x)\sum \limits _{m=0}^\infty \frac{1}{2^m}{\hat{\mu }}_{t_i}^h(m, \mathrm dx) = \int _{[0, T]}\int _{{\mathcal {X}}}f(x)\sum \limits _{m=0}^\infty \frac{1}{2^m}\Phi _h*\rho _t(m, \mathrm dx)\mathrm dt. \end{aligned}$$

We next note that $\sum \limits _{m=0}^\infty \frac{1}{2^m}{\hat{\mu }}_{t_i}^h = \Phi _h * {\hat{p}}_{t_i}$. Thanks to Corollary 6, under Assumption 4, $\sum \limits _{m=0}^\infty \frac{1}{2^m}\Phi _h * \rho _t(m, \mathrm dx) = \Phi _h * p_t(\mathrm dx)$, where *p* is the weak solution of the PDE (9) with initial condition $\mu _0$. Theorems 2.7, 2.9 of Lavenant et al. (2021) then apply, and allow us to conclude similarly to the proof of Theorem 2.3 of Lavenant et al. (2021). $\square$

**Fig. 2:**
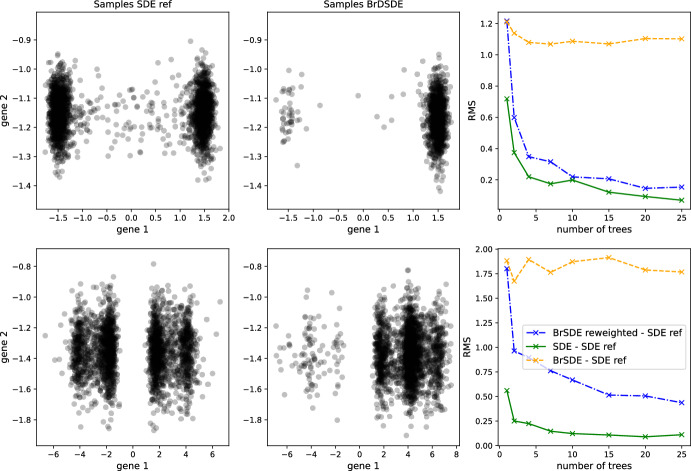
Numerical convergence of reweighted empirical measure associated to a branching SDE to the ground-truth distribution of the underlying SDE. First column: ground-truth distributions, obtained by simulating 2500 cells with the SDE. Second column: empirical measure obtained by simulating 25 trees containing, in total, 1050 leaves (first line) and 2584 leaves (second line), with the corresponding branching SDE. Third column: evolution of the RMS distance between the ground-truth and (blue) the reweighted empirical measure, (orange) the empirical measure (without reweighting), and (green) the empirical distribution obtained by simulating the non-branching SDE with the same number of cells as the number of leaves obtained with the branching SDE. Each row of plots correspond to one of the branching SDEs described in in Appendix 7

The convergence of the path measure in Theorem 7 strongly relies on the convergence of the reweighted empirical distributions to the temporal marginal probabilistic distributions of the SDE (1). Although a quantification of such convergence rate is beyond the scope of this article, in Fig. 2 we investigate numerically how the temporal marginal probabilistic distributions converge as the number *K* of observed trees increases. To do so, we simulate the long-time behavior of two branching SDEs, described fully in Appendix 7. Our accuracy metric is the root-mean-square (RMS) Energy Distance distance (Székely and Rizzo 2013) between the reweighted empirical distribution and the ground-truth distribution without branching (the latter computed by simulating thousands of cells under the underlying SDE). We plot the evolution of this distance as a function of the number of simulated trees used to form reweighted empirical distribution. As a reference point, for each *K* we simulate the same total number of cells using only the underlying SDE and we compare the RMS distance between the empirical distributions thus obtained and the ground-truth.

Because the reweighted distribution uses information on ${\mathcal {P}}({\mathbb {N}} \times {\mathcal {X}})$, which is a much bigger space than ${\mathcal {P}}({\mathcal {X}})$, it is *a priori* plausible that accurately reconstructing the temporal marginal probabilistic distributions requires much more data than in the case without branching. However, the results in the third column of Fig. 2 suggest that it is not the case: reweighting in these examples (blue line), although somewhat worse than using data directly from the underlying SDE (green line), significantly improves on not reweighting (orange line) as soon as $K \ge 2$.

For the second branching SDE, on the second row of Figure 2, the distance remains non-negligible out to 25 trees only because the branching rate is particularly high in a shallow well located around $x \approx 6$ in the first dimension. Thus, cells are likely to reach and stay in this well for the branching SDE but not for the underlying SDE. Reweighting by the generation numbers reduces the weight assigned to these cells, but requires more trees to fully adjust away the proliferation than are required for the branching SDE in the first line with only two wells.

The numerical evidence of Figure 2 suggests that using the reweighted distribution for trajectory inference is quite powerful in that it allows us to solve a problem in a very big space corresponding to branching SDEs with lineage tracing, while requiring a comparable amount of data to the simpler problem of trajectory inference for SDEs, posed in a much smaller space.

### Computational inference of trajectories and model characteristics

We are now going to illustrate how Theorem 7 can help to reconstruct the drift and the birth rate of a branching SDE, with examples from simulated datasets with lineage tracing. In subsection 4.2.1, we detail how to use an existing algorithm developed in Zhang et al. (2022) to reconstruct, from time-series of lineage-tracing single-cell data obtained from a branching SDE without death, the trajectories of a time-varying probabilistic distribution characterizing the process without proliferation. In subsection 4.2.2, we then propose an heuristic method to estimate from these trajectories the drift and the birth rate of the original process.

#### Trajectories of the time-varying distribution

We use the method described in Zhang et al. (2022) for reconstructing the trajectories of a time-varying probabilistic distribution from a time series of its temporal marginals. This algorithm optimizes a close variant of the functional $F_{N,\lambda ,h}$ used in the consistency result of Theorem 7. Explicitly, Chizat et al. propose finding

17 $$\begin{aligned} \inf \limits _{R \in {\mathcal {P}}({\mathcal {C}}([0, T], {\mathcal {X}}))}\left\{ \tau H(R | W^\tau ) + \frac{1}{\lambda }\sum _{i=1}^N \Delta t_i H\left( {\hat{\rho }}_{t_i} | \Phi _h * R_{t_i}\right) \right\} , \end{aligned}$$

where $({\hat{\rho }}_{t_i})$ is the sequence of empirical distributions describing the data at every timepoint. Their algorithm is described by a Mean-Field Langevin dynamics (Chizat 2022), and we refer to it as the *MFL algorithm* in the rest of the paper. In practice, it provides a sequence of *N* empirical probabilistic distributions (*N* sums of *M* Dirac point on ${\mathcal {X}}$), which they have shown to converge to the temporal marginal probabilistic distributions $\{R^*_{t_i}\}_{i=1,\cdots ,N}$ of the optimal path-measure $R^*$ corresponding to the unique solution of the problem (17) as $M \rightarrow \infty$. As the reweighted empirical measures defined in Eq. (15) are probabilistic distributions, we can directly apply the MFL algorithm, replacing at every $t_i$ the empirical probabilistic distribution ${\hat{\rho }}_{t_i}$ in (17) by the corresponding reweighted empirical distribution (15). We call this method the *reweighting method* in the following.

**Fig. 3:**
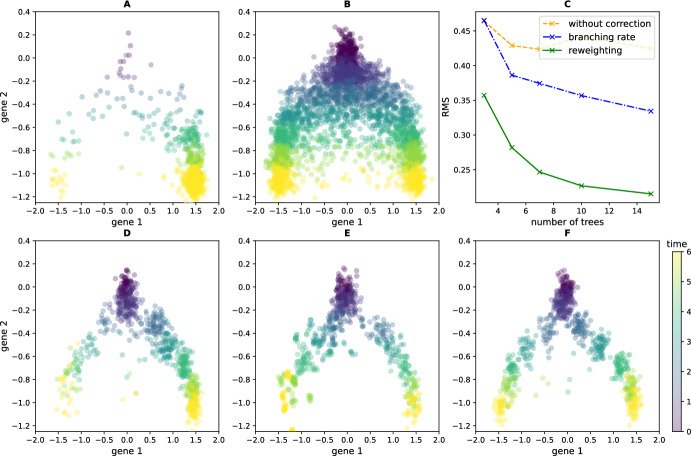
The time-varying distribution of an SDE with a double-well potential can be reconstructed using data from its associated branching SDE. Panel A shows the observed data when 5 independent trees are generated using potential $V_1$ from Appendix 7. B shows the ground-truth distribution we aim to recover, simulated using the underlying SDE with 500 cells at each timepoint. The cumulated RMS distance to this ground-truth at each timepoint is rather high if the MFL algorithm is applied with no correction for proliferation (C, dashed). Using a heuristic correction for known growth rates reduces the error (C, dashdotted), and applying our reweighting method reduces it further (C, solid line). The second row shows the reconstructed distributions using the three approaches: MFL without correction (D, visibly biased towards the more proliferative right well), MFL with the growth rate correction (E), and MFL on the reweighted marginal distributions (F)

Figure 3 illustrates the results of applying this method to datasets generated from a branching SDE with two wells (detailed as potential $V_1$ in Appendix 7). We compare three approaches: using no correction for proliferation (D), applying a heuristic correction ( Zhang et al. 2022, Section 4) that requires the branching rate to be known (E), and our new reweighting method (F). Reweighting significantly improves on both other strategies, even though the heuristic correction is given ground-truth branching rates that are never known in practice. The true branching rates are insufficient here because they only take into account the *average effect* of branching. In cases where sampling variation causes the ratio of cells in each well to differ from the expected average, using the branching rates directly biases the reconstruction. In contrast, our method uses the generation numbers to account for the exact effect of branching in each experimental realization.

This efficient reconstruction of the time-varying distribution associated to the underlying potential demonstrates the utility of the reweighting method and, more generally, of the information about generation numbers. As shown in the next sections, the recovered trajectory can subsequently be used to build an heuristic algorithm approximating the drift and birth rates that define the underlying branching SDE.

#### Estimation of the drift and branching rates

When there is no death, our reweighting method enables to build a sequence of *N* renormalized sum of *M* Dirac distributions, that we denote $\{\hat{P}^*_{t_i}\}_{i=1,\cdots ,N}$ that converge when $M\rightarrow \infty$ to the sequence of optimal temporal marginal probabilistic distributions $\{R^*_{t_i}\}_{i=1,\cdots ,N}$ corresponding to the time-varying probabilistic distribution $R^*$ of an optimal path-measure solution of the problem (17). Moreover, it has been proved in Zhang et al. (2022) that $R^*$ can be simply reconstructed from the optimal temporal marginal probabilistic distributions by simply solving a regularized optimal transport problem (with regularizer $\tau$) between each consecutive pair of them. We denote $\hat{P}^*$ the reconstructed empirical path-measure reconstructed in the same way from the sequence $\{\hat{P}^*_{t_i}\}_{i=1,\cdots ,N}$.

In addition, as mentioned in the introduction, it has been proved in Lavenant et al. (2021) that this optimal path-measure converges, when $N \rightarrow \infty$, to the one of the SDE described by the path-measure $P^\tau$ characterized by the same drift as the original branching SDE described by the path-measure *P* used to generate the data.

We are now going to use these results to propose an heuristic method for the estimation of the parameters of the original branching SDE described by *P*, *i.e* the branching rates $b^*$ and the drift $v^*$.

In a first place, let consider for simplicity that the parameter *M* used in the MFL algorithm is infinite, and that the reconstructed temporal marginal probabilistic distributions are then continuous. For $v^*$, we follow the proposal in Lavenant et al. (2021) to use the approximation

18 $$\begin{aligned} \forall i=1,\cdots ,T,\,\forall x \in {\mathcal {X}}:\, v^*(t_i, x) = {\textbf{E}}^{R^*}\left[ \frac{X_{t_{i+1}} - X_{t_i}}{t_{i+1} - t_i} \bigg | X_{t_i} = x \right] , \end{aligned}$$

which becomes exact in the limit $t_{i+1} - t_i \rightarrow 0$.

A similar way of characterizing an optimal birth rate $b^*$ would be to use the optimal time-varying distribution of the branching SDE, denoted $\rho ^{*}$. Indeed, provided that we have access to the optimal temporal marginal distributions of $\rho ^{*}$, we could then approximate:

19 $$\begin{aligned} \forall i=1,\cdots ,T,\,\forall x \in {\mathcal {X}}:\, b^*(t_i,x) \approx \frac{1}{t_{i+1} - t_i} \ln \left( {\textbf{E}}^{R^*}\left[ \frac{\rho ^{*}_{t_{i+1}}(X_{t_{i+1}})}{\rho ^{*}_{t_i}(X_{t_{i}})} \bigg | X_{t_i} = x\right] \right) , \end{aligned}$$

Considering that the optimal temporal marginals of $\rho ^{*}$ simply correspond to the sequence of observed empirical intensities $({\hat{\mu }}_{t_i})_{i=1\cdots ,n}$, we could estimate $b^*$ at each timepoint with formula (19).

However in practice we have only access to discrete path-measures, ${\hat{P}}^*$ instead of $R^*$ with our notations, and the second formula is not directly computable when $R^*$ is replaced by $\hat{P}^*$, since its support does not correspond with the support of the observed empirical distributions. Thus, in order to make the formula (19) usable, we have to estimate for each timepoint $t_i$ a new empirical distribution $\rho ^{*}_{t_i}$ having the same support as the empirical distribution $\hat{P}^*_{t_i}$. We start from the reweighted empirical distribution $\hat{\rho }_{t_i} = \sum \limits _m \frac{1}{2^m} {\hat{\mu }}_{t_i}(m)$, and propose the following two-step algorithm:

1. Find the optimal coupling $\pi ^*$ between the cells characterizing the two experimental distributions $\hat{P}^*_{t_i}$ and $\hat{\rho }_{t_i}$, by solving the optimal transport problem $W^2(\hat{P}^*_{t_i}, \hat{{\bar{\rho }}}_{t_i})$;
2. For every cell $x\in \text {Supp } \hat{P}^*_{t_i}$, we compute an associated generation number:
  $$\begin{aligned} m := \frac{\sum \limits _{(m', y) \in \text {Supp } {\hat{\mu }}_{t_i}} m' \pi ^*(x,y)}{\sum \limits _{y \in \text {Supp } {\hat{\mu }}_{t_i}}\pi ^*(x,y)}. \end{aligned}$$
  We then consider:
  20 $$\begin{aligned} \rho ^{*}_{t_i}(x) = 2^{m}\hat{P}^{*}_{t_i}(x). \end{aligned}$$

As an intuitive justification, it is easy to verify that if we do not use the MFL algorithm and simply set $\rho ^{*}_{t_i}(\cdot ) = \sum \limits _m \frac{1}{2^m} {\hat{\mu }}_{t_i}(m, \cdot )$, then $\pi ^*(x,y) = \delta _x(y)$ and we recover $\rho ^{*}_{t_i} = \sum \limits _m {\hat{\mu }}_{t_i}(m, \cdot )$.

**Fig. 4:**
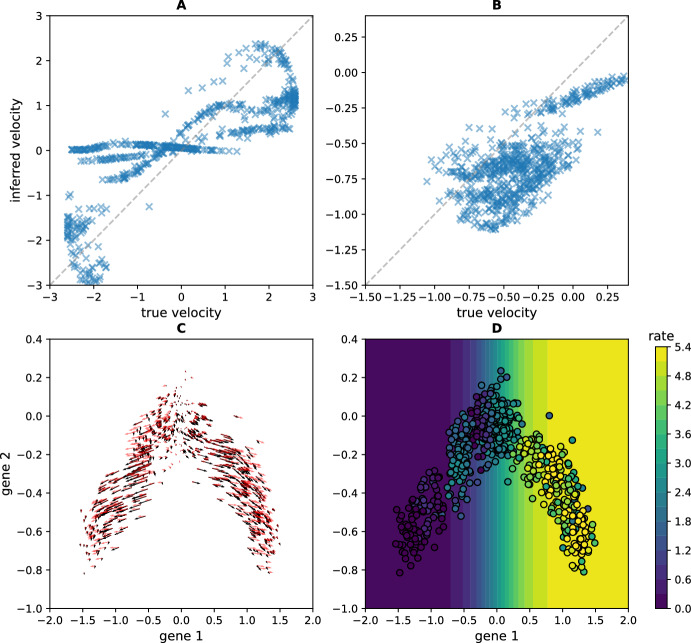
Estimation of (A)-(C) the velocity fields and (D) the birth rates reconstructed using the methods detailed in subsection 4.2.2. For the velocity fields, we represent in (A) (resp. B) the comparison between the inferred velocity field, with the formula (18) applied to the time-varying distribution reconstructed with the MFL algorithm (18), and the ground-truth using the parameters of the first branching SDE described in Appendix 7, for the first gene (resp. the second gene). The grey line represent the diagonal which would correspond to a perfect fit. In (C) we plot these two velocity fields along the trajectory, in dark for the inferred one and in red for the ground-truth. For the birth rate, we first use (20) to estimate from this distribution the new distribution with branching $\rho ^{*}$; second, we compute the associated birth rate using (19). The background of (D) corresponds to the true birth rate used for the simulation. The data corresponds to the reconstructed trajectories of 100 cells at each timepoints with the MFL algorithm from time-series of snapshots obtained by simulating 15 trees.

We apply these methods on the trajectories inferred in Fig. 3, and compare in Fig. 4 the estimated velocity field and birth rates with the simulation’s ground truth described in Appendix 7.

##### Remark 8

From a computational perspective, our algorithm introduces only minor additional operations on top of existing approaches, in particular the MFL algorithm. As a result, its computational limits are essentially the same, i.e. datasets of up to a few thousand cells in $\approx 10$ dimensions can be handled on a standard laptop. In practice, we therefore recommend applying the algorithm on the leading principal components of the dataset, after an initial PCA preprocessing step.

## Trajectory inference with death and subsampling

To be able to count a division event, we need to see at least one descendent of the two daughter cells. Thus, when cells can die, the generation number ${\tilde{m}}(X)$ observed with lineage tracing for a cell *X* at time *t* may differ from the real generation number *m*(*X*), because ${\tilde{m}}(X)$ only takes into account divisions for which both branches have a descendant alive at *t* (Figure 1). The reasoning of Section 4 is no longer valid. Subsampling causes an identical problem: if a division does not result in two branches that are subsampled at $t_i$, then it cannot be taken into account in ${\tilde{m}}(X)$. We consider these two situations together in this section. The case with only death or the case with only subsampling corresponds to setting $q(t_i) = 1$ or $d(t,x)=0$, respectively, in the results that follow.

The main analytical obstacle in the case with death is that the branching property (5) does not hold for the process on $({\tilde{m}}, x)$. In particular, the death of cell *x* not only removes *x* but also changes ${\tilde{m}}$ for every cell descended from the parent of *x*. Those interactions between cells introduce non-local terms in the master equation characterizing the time-varying distribution of the process, significantly complicating any calculations. Because of that complexity, we restrict the goals of this section to the following questions:

1. (Section 5.1) What bias results from applying the method in Section 4, *i.e* finding the probabilistic distribution minimizing a functional of the form (3) where the empirical distributions corresponds to the reweighted empirical distributions of ${\tilde{Z}}_t$?
2. (Section 5.2) How the information from *lineage tracing*, *i.e* the sequence of collection of lineage trees $\{{\mathcal {T}}_{t_i}^1,\cdots ,T_{t_i}^{K_i}\}_{i=1,\cdots ,N}$ and not only the generation numbers ${\tilde{m}}$, can be used to partially remove this bias?
3. (Section 5.3) Under what experimental conditions is the bias likely to be small?

We point out that while point 1. above is going to be treated rigorously, with Corollary 11 characterizing the aforementioned bias, points 2. and 3. are only going to be discussed in an heuristic way to provide both intuition and numerical evidence rather than formal proofs.

### Bias analysis

Using the same notation as in the previous sections for the process with real generation number *m*, we now write ${\tilde{Z}}_t({\tilde{m}})$ for the random variable describing a collection of tuples in ${\mathbb {N}} \times {\mathcal {X}}$ at time *t* observed after subsampling and let $\mathcal {{\tilde{N}}}(t):= \text {Supp}\,{\tilde{Z}}_t$. Similarly to $Z_t(m)$ defined in (7), $\tilde{Z}_t(\tilde{m})$ is a sum of Dirac masses corresponding to cells with observed generation number $\tilde{m}$. The law of ${\tilde{Z}}_t$ is described by a path-measure ${\tilde{P}} \in {\mathcal {P}}(\text {cdlg}([0, T], {\mathcal {M}}_+({\mathcal {X}})^{{\mathbb {N}}}))$. In this setting, ${\tilde{P}}$ does not satisfy the branching property defined above but a weaker property where we condition on each cell having at least one observed descendant:

- **The conditional branching property**: Let $t_i \in [0,T]$ and $t \le t_i$. For any $x \in {\mathcal {X}}$, we denote $\mathcal {{\tilde{N}}}_{x}(t):= \text {Supp}\,{\tilde{Z}}_t^{x}$, where ${\tilde{Z}}_t^{x}$ is the random variable describing the collection of tuples at time *t* descending from the same common ancestor in *x* at time 0. For any sum of Dirac masses $\mu$ in ${\mathbb {N}} \times {\mathcal {X}}$ and any collection of measurable functions $\{u(m)\}_{m\in {\mathbb {N}}}$ from ${\mathcal {X}}$ to ${\mathbb {R}}^+ \cup \{+\infty \}$, we have:
  21 $$\begin{aligned} {\textbf{E}}^{{\tilde{P}}}_{\mu }\bigg [\prod \limits _{({\tilde{M}},X) \in \mathcal {{\tilde{N}}}(t)}&u({\tilde{M}}, X) \bigg |\, \forall ({\tilde{m}},x) \in \text {Supp}\,\mu ,\,\big |\mathcal {{\tilde{N}}}_{ x}(t_i)| \ge 1 \bigg ] = \nonumber \\&\prod \limits _{({\tilde{m}},x) \in \text {Supp}\,\mu } {\textbf{E}}^{{\tilde{P}}}_{({\tilde{m}},x)}\bigg [\prod \limits _{({\tilde{M}}, X) \in \mathcal {{\tilde{N}}}(t)}u({\tilde{M}}, X)\bigg |\, |\mathcal {{\tilde{N}}}(t_i)| \ge 1\bigg ]. \end{aligned}$$

This equality is immediate for the branching SDE without generation numbers because of the independence of branches stated by the branching property. For the branching SDE with observable generation numbers, each branch starting from a cell in the initial distribution evolves in ${\mathbb {N}} \times {\mathcal {X}}$ independently from the others conditional on the fact that all these branches survive, *i.e* that each cell in the initial distribution has at least one subsampled descendant. Indeed, the only event in a branch that can impact the other branches is its extinction, which would update the observable generation numbers of cells in other branches. The initial branches are therefore independent conditional on none of them going extinct, as expressed by (21).

Now, for every timepoint $t_i \in [0,T]$, we consider a new collection of random variables $({\tilde{Z}}_t^{t_i}({\tilde{m}}))_{m \in {\mathbb {N}}}$, describing at every earlier time $t \in [0, t_i]$ the collection of tuples that have at least one subsampled descendant at time $t_i$. We denote $(\mathcal {{\tilde{F}}}^{t_i}_t)_{t \in [0, t_i]}$ the natural filtration associated to this stochastic process. The conditional branching property (21) holds for this new process as well.

For each family, we define the collection of time-varying distributions $(\{\rho ^{t_i}({\tilde{m}})\}_{m\in {\mathbb {N}}})_t$, by:

22 $$\begin{aligned} \forall {\tilde{m}} \ge 0,\,\forall t \le t_i:\, \rho _t^{t_i}({\tilde{m}}):\, A \rightarrow {\textbf{E}}^{{\tilde{P}}}\big [{\tilde{Z}}_t^{t_i}({\tilde{m}},A)\big |\, |\mathcal {{\tilde{N}}}(t_i)| \ge 1 \big ]. \end{aligned}$$

Importantly, at each measurement time $t_i$, the new random variables coincide with our observations: ${\tilde{Z}}_{t_i}^{t_i}({\tilde{m}}) = {\tilde{Z}}_{t_i}({\tilde{m}})$. This means that the collection of marginal distributions $\{\rho ^{t_i}_{t_i}({\tilde{m}})\}$ coincides at time $t_i$ with the collection of temporal marginals of the path-measure ${\tilde{P}}_{t_i}$ that describes the branching SDE with observable generation numbers, conditional on subsampling at least one cell at time $t_i$.

The importance of conditioning on descendants being subsampled leads us to define a branch survival probability:

23 $$\begin{aligned} S^{t_i}:\,(t,x)\rightarrow {\textbf{P}}_x(|\mathcal {{\tilde{N}}}(t_i-t)| \ge 1). \end{aligned}$$

We can now state the main result of this section:

#### Proposition 9

Let $t_i \in [0, T]$. Under Assumption (3) and assuming that the branch survival probability $S^{t_i}(\cdot , x)$ is continuous for all $x \in {\mathcal {X}}$ and $S^{t_i}(t, \cdot ) \in C^1({\mathcal {X}})$ for all $t \in [0,t_i]$, the collection of time-varying distributions $\{\rho ^{t_i}({\tilde{m}})\}_{{\tilde{m}}\in {\mathbb {N}}}$ defined by (22) solves the following system of PDEs in the weak sense:

24 $$\begin{aligned} \forall {\tilde{m}} \ge 0:\, \partial _t \rho ^{t_i}({\tilde{m}}) = {\mathcal {L}}^{*}_{\Psi - \tau \ln S^{t_i}, \tau }\rho ^{t_i}({\tilde{m}}) + 2bS^{t_i} \rho ^{t_i}({\tilde{m}}-1) - b S^{t_i}\rho ^{t_i}({\tilde{m}}), \end{aligned}$$

with initial condition $\rho ^{t_i}_0({\tilde{m}}, \cdot ) \propto {\mu _0 S^{t_i}(0, \cdot )1\!\!1_{{\tilde{m}} = 0}}$.

Note that this system of PDE coincides with Eq. (11) for new branching SDE with a modified potential $\Psi - \tau \ln S^{t_i}$, a modified birth rate $bS^{t_i}$, and no death. In the following, we will call the quantity

25 $$\begin{aligned} \Psi ^{t_i} := -\tau \ln S^{t_i}, \end{aligned}$$

the *bias* in the potential; $-\nabla \Psi ^{t_i} = \tau \nabla \ln S^{t_i}$ is then the bias in the drift.

#### Remark 10

Ideally, the regularity assumptions on the function $S^{t_i}$ in Proposition 24 should be derived directly from the properties of the branching SDE, namely from the regularity of the branching rates and of the potential function. Addressing this issue, however, lies beyond the scope of the present work and is left for future theoretical investigations.

#### Proof

The proof of this proposition follows the same steps as the proof of (11), using the Markov property, invariance by translation, and the conditional branching property. In particular, by the conditional branching property, it is enough to prove the proposition starting from a cell in an arbitrary tuple $({\tilde{m}},x) \in {\mathbb {N}} \times {\mathcal {X}}$. At a small time $\delta t$, the set of complementary events that we consider are now:

- $A^b_2(\delta t)$: the cell has divided into two cells and the two cells each have a surviving descendant at *T*;
- $A^b_1(\delta t)$: the cell has divided into two cells and only one cell has a surviving descendant at *T*;
- $A^b_0(\delta (t)$: the cell has divided into two cells and no cell has a surviving descendant at *T*;
- $A^d(\delta t)$: the cell is dead;
- $A^c(\delta t)$: the cell has neither divided nor died.

We denote $$u(t_i, t, {\tilde{m}}, x) = {\textbf{E}}_{({\tilde{m}}, x)}\left[ \prod \limits _{\begin{array}{c} ({\tilde{M}}, X) \in \mathcal {{\tilde{N}}}(\delta t) \\ \mathcal {{\tilde{N}}}_{X}(t_i-t) \ge 1 \end{array}} u_0({\tilde{M}},X) \bigg | |\mathcal {{\tilde{N}}}(t_i)| \ge 1\right] $$, where the expected value is taken under the law of ${\tilde{P}}$. It is clear that the probability of $A^b_0$ and $A^d$ are 0 conditional on $\{|\mathcal {{\tilde{N}}}(t_i)| \ge 1\}$, and that the probability of $\{|\mathcal {{\tilde{N}}}(t_i)| \ge 1\}$ is 1 conditionally to $A^b_2(\delta t)$ and $A^b_1(\delta t)$. We have then:

$$\begin{aligned} u(t_i, \delta t, {\tilde{m}}, x)&= (1 - {\textbf{P}}_{x}(A^b_2(\delta t) \cup A^b_1(\delta t)\big | |\mathcal {{\tilde{N}}}(t_i)| \ge 1)) \times \\ &{\textbf{E}}_{({\tilde{m}}, x)}\left[ \prod \limits _{\begin{array}{c} ({\tilde{M}}, X) \in \mathcal {{\tilde{N}}}(\delta t) \\ \mathcal {{\tilde{N}}}_{X}(t_i-t) \ge 1 \end{array}} u_0({\tilde{M}},X) \bigg | (|\mathcal {{\tilde{N}}}(t_i)| \ge 1) \cap A^c\right] \\ &+ {\textbf{P}}_{x}(A^b_2(\delta t)\big | |\mathcal {{\tilde{N}}}(t_i)| \ge 1)\times {\textbf{E}}_{({\tilde{m}}, x)}\left[ \prod \limits _{\begin{array}{c} ({\tilde{M}}, X) \in \mathcal {{\tilde{N}}}(\delta t) \\ \mathcal {{\tilde{N}}}_{X}(t_i-t) \ge 1 \end{array}} u_0({\tilde{M}},X) \bigg | A^b_2(\delta t) \right] \\ &+ {\textbf{P}}_{x}(A^b_1(\delta t)\big | |\mathcal {{\tilde{N}}}(t_i)| \ge 1)\times {\textbf{E}}_{({\tilde{m}}, x)}\left[ \prod \limits _{\begin{array}{c} ({\tilde{M}}, X) \in \mathcal {{\tilde{N}}}(\delta t) \\ \mathcal {{\tilde{N}}}_{X}(t_i-t) \ge 1 \end{array}} u_0({\tilde{M}},X) \bigg | A^b_1(\delta t)\right] . \end{aligned}$$

Moreover, using Bayes’ law and a little algebra, we have:

$$\begin{aligned} {\textbf{P}}_{x}(A^b_2(\delta t) \cup A^b_1(\delta t)\big | |\mathcal {{\tilde{N}}}(t_i)| \ge 1)&= {\textbf{P}}_{x}(A^b_2(\delta t) \cup A^b_1(\delta t)) \\&\times \frac{{\textbf{P}}_{x}(|\mathcal {{\tilde{N}}}(t_i)| \ge 1\big | A^b_2(\delta t) \cup A^b_1(\delta t))}{{\textbf{P}}_{x}(|\mathcal {{\tilde{N}}}(t_i)| \ge 1)},\\ {\textbf{P}}_{x}(A^b_2(\delta t)\big | |\mathcal {{\tilde{N}}}(t_i)| \ge 1)&= {\textbf{P}}_{x}(A^b_2(\delta t) \cup A^b_1(\delta t)\big | |\mathcal {{\tilde{N}}}(t_i)| \ge 1) \\&\times \frac{{\textbf{P}}_{x}(|\mathcal {{\tilde{N}}}(t_i - \delta t)| \ge 1)^2}{{\textbf{P}}_{x}(|\mathcal {{\tilde{N}}}(t_i - \delta t)| \ge 1\big | A^b_2(\delta t) \cup A^b_1(\delta t))},\\&{\textbf{P}}_{x}(A^b_1(\delta t)\big | |\mathcal {{\tilde{N}}}(t_i)| \ge 1) = {\textbf{P}}_{x}(A^b_2(\delta t) \cup A^b_1(\delta t)\big | |\mathcal {{\tilde{N}}}(t_i)| \ge 1) \\ &\times \frac{2{\textbf{P}}_{x}(|\mathcal {{\tilde{N}}}(t_i - \delta t)| \ge 1) (1-{\textbf{P}}_{x}(|\mathcal {{\tilde{N}}}(t_i - \delta t)| \ge 1))}{{\textbf{P}}_{x}(|\mathcal {{\tilde{N}}}(t_i - \delta t)| \ge 1\big | A^b_2(\delta t) \cup A^b_1(\delta t))}. \end{aligned}$$

Note that these quantities are well defined since under Assumption 3 the function $S^{t_i}(t, \cdot )$ is strictly positive for all *t*. We then obtain by continuity of $S^{t_i}(\cdot , x)$:

$$\begin{aligned}&\lim \limits _{\delta t \rightarrow 0}\frac{{\textbf{P}}_{x}(A^b_2(\delta t) \cup A^b_1(\delta t)\big | |\mathcal {{\tilde{N}}}(t_i)| \ge 1)}{\delta t} = b (2 - S^{t_i}(0,x)),\\&\lim \limits _{\delta t \rightarrow 0}\frac{{\textbf{P}}_{x}(A^b_2(\delta t)\big | |\mathcal {{\tilde{N}}}(t_i)| \ge 1)}{\delta t} = b S^{t_i}(0,x),\\&\lim \limits _{\delta t \rightarrow 0}\frac{{\textbf{P}}_{x}(A^b_1(\delta t)\big | |\mathcal {{\tilde{N}}}(t_i)| \ge 1)}{\delta t} = 2b (1 - S^{t_i}(0,x)). \end{aligned}$$

Finally, thanks to the conditional branching property and following the same reasoning as in the proof of (11), Step 1.:

$$\begin{aligned} \lim \limits _{\delta t \rightarrow 0} \frac{u(t_i, \delta t, {\tilde{m}}, x) - u_0({\tilde{m}}, x)}{\delta t} =&\lim \limits _{\delta t \rightarrow 0} \frac{{\textbf{E}}_x^p\left[ u_0({\tilde{m}}, X_{\delta t})\bigg | |\mathcal {{\tilde{N}}}(t_i)| \ge 1\right] - u_0({\tilde{m}}, x)}{\delta t} \\ &- b S^{t_i}(0,x) u_0({\tilde{m}}, x) + b S^{t_i}(0,x) u_0({\tilde{m}}+1, x)^2, \end{aligned}$$

where ${\textbf{E}}_x^p$ denotes the expected value under the law of the underlying branching-free SDE (1). To find the value of the right-hand side term in the first line, we use the Itô formula, which states that for all ${\tilde{m}},x$:

$$\begin{aligned} u_0({\tilde{m}}, X_{\delta t}) = u_0({\tilde{m}}, x) + \int _0^{\delta t} {\mathcal {L}}_{\Psi , \tau } u_0({\tilde{m}}, X_s)\mathrm ds + \tau \int _0^{\delta t} \nabla u_0({\tilde{m}}, X_s)\mathrm dB_s, \end{aligned}$$

ensuring that, as long as $u_0 \in C^2_b({\mathcal {X}})$:

$$\begin{aligned}&\lim \limits _{\delta t \rightarrow 0} \frac{{\textbf{E}}_x^p\left[ u_0({\tilde{m}}, X_{\delta t})\bigg | |\mathcal {{\tilde{N}}}(t_i)| \ge 1\right] - u_0({\tilde{m}}, x)}{\delta t} = {\mathcal {L}}_{\Psi , \tau } u_0({\tilde{m}}, x) \\&\quad +\tau \sum \limits _{j=1}^g \partial _{x_j} u_0({\tilde{m}}, x) \lim \limits _{\delta t \rightarrow 0} \frac{1}{\delta t}{\textbf{E}}_{x}\left[ B_{\delta t}^j - x_j\bigg | |\mathcal {{\tilde{N}}}(t_i)| \ge 1\right] , \end{aligned}$$

where $\{B_{\delta t}^1,\cdots ,B_{\delta t}^g\}$ are a collection of *g* independent random variables describing Brownian motions at time $\delta _t$.

Using Bayes’ rule once again, we have in every direction $j = 1,\cdots ,g$:

26 $$\begin{aligned} \lim \limits _{\delta t \rightarrow 0} \frac{1}{\delta t}{\textbf{E}}_{x}\left[ B_{\delta t}^j - x_j\bigg | |\mathcal {{\tilde{N}}}(t_i)| \ge 1\right] =&\lim \limits _{\delta t \rightarrow 0} {\textbf{E}}_{N(0, \frac{1}{\delta t})} \left[ {Y}\frac{{\textbf{P}}_{x + \delta tYe_j - \delta t\nabla \Psi (t, x)}(|\mathcal {{\tilde{N}}}(t_i - \delta t)| \ge 1)}{{\textbf{P}}_x(|\mathcal {{\tilde{N}}}(t_i)| \ge 1)} \right] ,\nonumber \\ =&\lim \limits _{\delta t \rightarrow 0} {\textbf{E}}_{N(0, \frac{1}{\delta t})} \left[ {Y}\frac{S^{t_i}(\delta t, x + \delta t Y e_j - \delta t\nabla \Psi (t, x))}{S^{t_i}(0, x)} \right] . \end{aligned}$$

Using the fact that $S^{t_i}$ is differentiable w.r.t *x*, we can use its Taylor expansion in every direction $j=1,\cdots ,g$ to obtain:

$$\begin{aligned}&{\textbf{E}}_{N(0, \frac{1}{\delta t})} \left[ {Y}\frac{S^{t_i}(\delta t, x + \delta t Y e_j)}{S^{t_i}(0, x)} \right] = {\textbf{E}}_{N(0, \frac{1}{\delta t})} \\&\quad \left[ {Y} \frac{S^{t_i}(\delta t, x)}{S^{t_i}(0, x)} + (Y^2\delta t - Y\delta t \nabla \Psi (t, x)) \frac{\partial _{x_j}S^{t_i}(\delta t, x)}{S^{t_i}(0, x)} + O(Y^3 \delta t^2)\right] \\&\quad = \frac{\partial _{x_j}S^{t_i}(\delta t, x)}{S^{t_i}(0, x)} + O(\delta t). \end{aligned}$$

The remainder is $O(Y^4\delta t^3) = O(\delta t)$ because the symmetry of the distribution of *Y* makes the odd terms in the Taylor expansion vanish. By continuity of $\nabla S^{t_i}$ w.r.t *t*, the limit (26) is then equal to $\partial _{x_j} \ln S^{t_i}(0, x)$.

Thus we obtain that $\forall {\tilde{m}} \in {\mathbb {N}}, \forall x \in {\mathcal {X}}$:

$$\begin{aligned} \frac{\mathrm d}{\mathrm d t}u(t_i, t, {\tilde{m}}, x)\big |_{t = 0}&= {\mathcal {L}}_{\Psi + \Psi ^{t_i}, \tau } u_0({\tilde{m}},x) + bS^{t_i} u_0({\tilde{m}}+1, x)^2 - bS^{t_i} u_0({\tilde{m}}, x),\\ &:= \mathcal {{\tilde{K}}}_{\Psi , \tau } u_0. \end{aligned}$$

We can now reason in a way very similar to Step 2. of the proof of (11), using the Markov property and the conditional branching property. Indeed, we have for all $t, s > 0$ such that $t+s \le t_i$:

$$\begin{aligned} u&(t+s, {\tilde{m}}, x) = {\textbf{E}}_{({\tilde{m}}, x)}\left[ {\textbf{E}}_{({\tilde{m}}, x)}\left[ \prod \limits _{\begin{array}{c} ({\tilde{M}}, X) \in \mathcal {{\tilde{N}}}(t+s) \\ \mathcal {{\tilde{N}}}_{X}(t_i-t-s) \ge 1 \end{array}} u_0({\tilde{M}},X) \big | \left( |\mathcal {{\tilde{N}}}(t_i)| \ge 1\right) \cap \mathcal {{\tilde{F}}}^{t_i}_s \right] \Bigg | |\mathcal {{\tilde{N}}}(t_i)| \ge 1\right] , \\&= {\textbf{E}}_{({\tilde{m}}, x)}\left[ {\textbf{E}}_{{\tilde{Z}}_s^{t_i}}\left[ \prod \limits _{\begin{array}{c} ({\tilde{M}},X) \in \mathcal {{\tilde{N}}}(t) \\ \mathcal {{\tilde{N}}}_{X}(t_i - t-s) \ge 1 \end{array}}u_0({\tilde{M}}, X) \bigg |\, \forall ({\tilde{M}}',X') \in \text {Supp}\,{\tilde{Z}}_s^{t_i},\,\big |\mathcal {{\tilde{N}}}_{X'}(t_i-s)| \ge 1\right] \Bigg | |\right. \\&\quad \qquad \qquad \left. \mathcal {{\tilde{N}}}(t_i)| \ge 1\right] , \\&= {\textbf{E}}_{({\tilde{m}}, x)}\left[ \prod \limits _{\begin{array}{c} ({\tilde{M}}', X') \in \mathcal {{\tilde{N}}}(s) \\ \mathcal {{\tilde{N}}}_{X'}(t_i-s) \ge 1 \end{array}} {\textbf{E}}_{({\tilde{M}},X)}\left[ \prod \limits _{\begin{array}{c} ({\tilde{M}}, X) \in \mathcal {{\tilde{N}}}(t) \\ \mathcal {{\tilde{N}}}_{X'}(t_i - t-s) \ge 1 \end{array}}u_0({\tilde{M}}, X)\bigg |\, |\mathcal {{\tilde{N}}}(t_i-s)| \ge 1\right] \Bigg | |\mathcal {{\tilde{N}}}(t_i)| \ge 1 \right] ,\\&= {\textbf{E}}_{({\tilde{m}}, x)}\left[ \prod \limits _{\begin{array}{c} ({\tilde{M}}', X') \in \mathcal {{\tilde{N}}}(s) \\ \mathcal {{\tilde{N}}}_{X'}(t_i-s) \ge 1 \end{array}} u(t_i - s, t, {\tilde{M}}', X')\bigg | |\mathcal {{\tilde{N}}}(t_i)| \ge 1 \right] . \end{aligned}$$

Thanks to Step 1, we obtain the following system of PDEs:

$$\begin{aligned} \forall {\tilde{m}} \in {\mathbb {N}}, \forall x \in {\mathcal {X}}:\, \frac{\mathrm d}{\mathrm d s}u(t_i, t+s, {\tilde{m}}, x)\big |_{s = 0} = \mathcal {{\tilde{K}}}_{\Psi , \tau } u(t_i, t, {\tilde{m}}, x). \end{aligned}$$

Finally, Step 3. of the proof of Proposition 5 can be repeated without any change, and the initial condition follows directly from the definition of $\rho ^{t_i}$, in (22), using Bayes’ rule. $\square$

We can now state our main result for the case with death and subsampling. Importantly, this result makes explicit how the fact that we observe only cells that have survived biases both the effective drift and the initial distribution of the inferred dynamics.

#### Corollary 11

Under the assumptions of Proposition 9, at each observation time $t_i$, the reweighted distribution

$$ \bar{\rho }_{t_i} := \sum _{\tilde{m} \ge 0} \frac{1}{2^{\tilde{m}}} \rho _{t_i}(\tilde{m}) $$

coincides with $\rho _{t_i}$, where $(\rho _t)_{t \in [0,t_i]}$ is the solution of the following PDE:

27 $$\begin{aligned} \partial _t \rho = {\mathcal {L}}^*_{\Psi + \Psi ^{t_i}, \tau } \rho , \end{aligned}$$

with initial condition $\rho _0(\cdot ) \propto \mu _0(\cdot ) S^{t_i}(0, \cdot )$, where the bias potential $\Psi ^{t_i}$ is defined in (25).

#### Proof

The initial condition follows directly from Proposition 9. At each observation time $t_i$, we have by construction that $\tilde{Z}^{t_i}_{t_i}(\tilde{m}) = \tilde{Z}_{t_i}(\tilde{m})$, which implies that the collections of intensity measures

$$ \{\rho ^{t_i}_{t_i}(\tilde{m})\}_{\tilde{m} \in \mathbb {N}} \quad \text {and} \quad \{\rho _{t_i}(\tilde{m})\}_{\tilde{m} \in \mathbb {N}} $$

coincide at time $t_i$. The result then follows by applying the same argument as in the proof of Corollary 6 to the system of PDEs (24). $\square$

Corollary 11 allows us to clarify the path-measure that is implicitly reconstructed when applying the reweighting method described in Section 4.2.1 to scRNA-seq datasets with lineage tracing in the presence of both death and subsampling. To make this connection explicit, consider the case where measurements are performed at only two timepoints $$t_1 = 0$$ and $$t_2 = T$$. The corollary shows that the reweighted distributions then correspond to $$\mu _0$$ at time 0 and to $$p_T$$ at time *T*, where $$p_T$$ is the solution at time *T* of the PDE (27) associated with an SDE with drift $$-\nabla (\Psi + \Psi ^{T})$$ and initial condition proportional to $$\mu _0(\cdot ) S^{T}(0,\cdot )$$. Since the drift of this effective dynamics remains a gradient, the assumptions of Theorem 2.3 in Lavenant et al. (2021) are satisfied. As a result, solving the Schrödinger problem between the distributions $$\mu _0(\cdot ) S^{T}(0,\cdot )$$ at time 0 and $$ p_T$$ at time *T* yields a time-varying distribution that coincides with that of the biased SDE (27), at least when $$T \rightarrow 0$$.

#### Remark 12

Note that in typical experimental settings, it is reasonable to approximate $S^{t_i}(0,x) \approx 1$ for $x \in \operatorname {supp}(\mu _0)$. Indeed, biological measurements are usually performed on growing tissues, where the net proliferation rate $(b-d)$ is high at small times, close to the cell of origin. In such regimes, the probability of complete extinction before observation is small at moderate time *T*, especially when starting from regions where $\mu _0$ has significant mass. Under this approximation, the modified initial condition $\mu _0(\cdot ) S^{T}(0,\cdot )$ remains close to $\mu _0$, and solving the Schrödinger problem between $\mu _0$, ignoring $S^{T}(0)$, and the reweighted distributions $p_{T}$ is expected to yield a reconstruction of a gradient drift that is close to the true biased drift. It is nevertheless important to remind that $S^{T}(0)$ necessarily differs (even slightly) from 1 as soon as the death rate *d* is not uniformly 0, and that ignoring this term in the initial condition introduces an additional source of bias that should be taken into account in the analysis, which we leave for future work.

When the measurements are done at many timepoints $(t_i)_{i=1,\cdots ,N}$, at each timepoint $t_i$ the reweighted empirical distribution ${\hat{p}}_{t_i}$ is associated to the temporal marginal probabilistic distribution $p_{t_i}$ of an SDE with a timepoint-specific drift bias $-\nabla \Psi ^{t_i}$. In other words, the correction term $\Psi ^{t_i}$ depends explicitly on the chosen snapshot $t_i$, so that no single biased SDE can simultaneously interpolate all observed distributions. This motivates the following definition: we say that a collection of marginal probabilistic distributions $\{p_{t_i}\}_{i=1}^N$ is *compatible* with a given drift if the temporal marginal distributions of the resulting SDE coincide with $\{p_{t_i}\}_{i=1}^N$. In the case with death and subsampling, the marginal probabilistic distributions obtained from reweighting are not compatible in this sense for the drift bias $\nabla \Psi ^{t_i}$ defined in Proposition 9, since each $p_{t_i}$ corresponds to a different effective bias. Whether a drift making the collection of reweighted marginal distributions compatible can be gradient or not with more than two timepoints remains unclear.

**Fig. 5:**
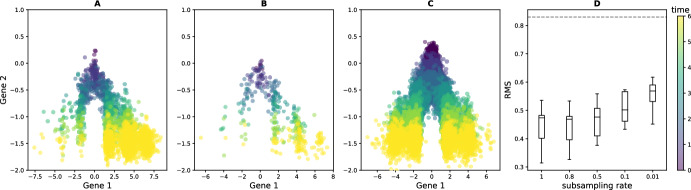
Evolution of the bias w.r.t the subsampling rate. Simulations of the second branching SDE described in Appendix 7 (A) without subsampling, (B) subsampling with a decreasing rate from 1 to 0.05, and (C) only the SDE without branching. In (D), we compare the cumulated RMS distance between the reweighted time-varying distributions and the ground-truth SDE at each timepoint for 5 different decreasing sequences of subsampling rates (including $q(t)=1$ where no subsampling occurs). Each box and whisker plot shows the distribution of RMS distances from 10 independent simulations. The subsampling rate indicated is *q*(*T*), the rate for the last timepoint. The horizontal dashed line corresponds to the cumulated RMS distance between the simulations in (C) and the simulations of (A) without reweighting.

Our numerical experiments suggest the bias part of this unknown drift, while not fully characterized, will be small in practice. Fig. 5 presents the evolution of the bias arising in the reconstruction of the time-varying distribution of the underlying SDE as the subsampling rate increases. Although, as expected, the quality of the reconstruction decreases with increasing subsampling rate, the loss in accuracy due to the subsampling is significantly smaller than the gain from reweighting. The cumulated RMS distance when reweighting after subsampling with $q(T) = 0.01$ (Fig. 5F, right) is 0.582, compared to 0.473 when reweighting with no subsampling (Fig. 5F, left) and 0.823 without reweighting (Fig. 5F, upper dashed line). The relatively small bias is not specific to the choice of the branching SDE’s parameters: in our numerical experiments, we found it difficult to choose a potential, an associated branching rate, and a subsampling rate such that i) the trajectories show biologically meaningful structure, ii) the extinction event is unlikely, and iii) the bias is significant. In particular, we chose the more complex potential function of the two in Fig. 2 for the simulations of Fig. 5 because the effect of subsampling was not visible with the simpler two-well potential.

The main theoretical reason for this bias to be that small is that it is scaled by the diffusion coefficient $\tau$. In our simulations, even when significant differences exist in the branching rate between the potential wells, increasing $\tau$ causes the stochasticity from diffusion to erase the structure of the potential before it makes the drift bias $-\nabla \Psi ^{t_i} (= \tau \nabla \ln S^{t_i})$ substantial. We discuss this relation further in Section 5.3.

While the bias may rarely be large, controlling it would improve inference accuracy. To do so, the next section introduces an heuristic method for removing the incompatibility between reweighted marginal distributions by using more information from each tree ${\mathcal {T}}_i$ than the observable generation numbers we have restricted ourselves to thus far. More precisely, we are going to build form the sequence of collection of lineage trees $\{\mathcal {T}_{t_i}^1,\cdots ,\mathcal {T}_{t_i}^{K_i}\}_{i=1,\cdots ,N}$ a sequence of pairs of empirical probabilistic distributions $(\hat{p}_{t_i}^s,\hat{p}_{t_{i+1}})_{i=1,\cdots ,N}$ that are compatible for the drift $-\nabla (\Psi + \Psi ^{t_{i+1}})$.

### Controlling the bias from death and subsampling

The starting point of the method is that for any pair of timepoints $(t_i, t_{i+1})$, we would like to interpret the collection of marginal distributions observed at $t_{i+1}$, $\{{\rho }_{t_{i+1}}(\tilde{m})\}_{\tilde{m} \in {\mathbb {N}}}$, as the solution at time $t_{i+1} - t_i$ of a system of master equations of the form (24) (so with only proliferation and drift $-\nabla \left( \Psi + \Psi ^{t_{i+1}}\right)$) starting at some collection of marginal distributions at $t_i$. Clearly, this is not the case for the collection of marginal distributions observed at $t_i$, $\{\rho _{t_i}(m)\}_{m \in {\mathbb {N}}}$, each cell alive at time $t_{i}$ would have at least one descendant subsampled at $t_{i+1}$ because it is quite possible in general for branches to die out between $t_i$ and $t_{i+1}$, in which case some cells observed at $t_i$ do not correspond to the ancestor of any cell at $t_{i+1}$. Our aim is thus to reconstruct a collection of marginal distributions $\{\rho _{t_i}^s(\tilde{m})\}_{\tilde{m} \in {\mathbb {N}}}$ describing the cells sampled at time $t_i$ with at least one observed descendant at time $t_{i+1}$. Indeed, from Corollary 11, the pair of associated reweighted marginal distribution $(p_{t_i}^s, p_{t_{i+1}})$ would be compatible for the SDE with drift $-\nabla \left( \Psi + \Psi ^{t_{i+1}}\right)$.

Thus, the first step is estimating the collection of marginal distributions $\{\rho _{t_i}^s(\tilde{m})\}_{\tilde{m} \in {\mathbb {N}}}$ from the observations, using more information about the lineage tree than the observable generation numbers that we have used until now. For this sake, we propose a two-step algorithm inspired from LineageOT (Forrow and Schiebinger 2021):

1. For every $\tilde{m} \in {\mathbb {N}}$, make an initial estimate of $\rho _{t_i}^s(\tilde{m}, {\mathcal {X}})$ using uniquely the collection of lineage tree $\{\mathcal {T}_{t_{i+1}}^{1},\cdots ,\mathcal {T}_{t_{i+1}}^{K_{i+1}}\}$, by going backward from $t_{i+1}$ to $t_i$ via a graphical model to estimate the position in ${\mathcal {X}}$ of every cell with a generation number equal to ${\tilde{m}}$ at $t_i$ (according to the structure of ${\mathcal {T}}_{t_{i+1}}$). This step is performed in a similar fashion to the ancestor estimation step of LineageOT;
2. Improve the estimate of the collection of marginal distributions $\{\rho _{t_i}^s(\tilde{m})\}_{\tilde{m} \in {\mathbb {N}}}$ by matching the estimation of step 1 with the observed collection of marginal distributions observed at time *i*, $\{\rho _{t_i}(\tilde{m})\}_{\tilde{m} \in {\mathbb {N}}}$, using optimal transport, in a similar fashion to the matching step of LineageOT.

**Fig. 6:**
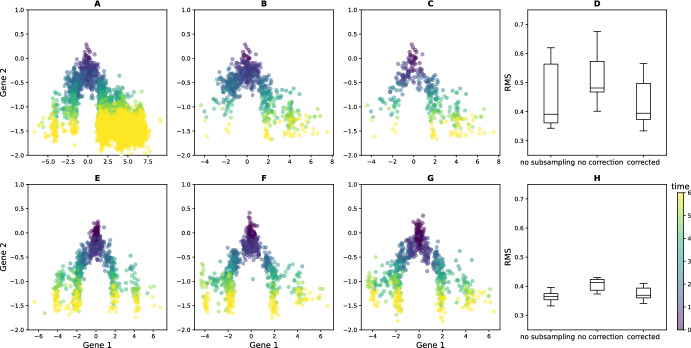
Representation of the framework described in Sections 5.2 to build for each pair of timepoints $(t_i, t_{i+1})$, pairs of *compatible* distributions $({\hat{p}}^s_{t_i}, {\hat{p}}_{t_{i+1}})$ in the sense that they correspond to the temporal marginal probabilistic distributions of an underlying SDE whose drift is known at the two timepoints (represented by a green square in the figure). First, we build at each timepoint $t_i$ the *reweighted marginal probablistic distribution* ${\hat{p}}_{t_i}$ from the observed collection of marginal distributions $\{{\hat{\rho }}_{t_i}({\tilde{m}})\}_{{\tilde{m}} \ge 0}$, which corresponds to the temporal marginal probabilistic distributions of a SDE with timepoint-specific drift $-\nabla \left( \Psi + \Psi ^{t_i}\right)$. As the pair $({\hat{p}}_{t_i}, {\hat{p}}_{t_{i+1}})$ is not compatible (represented by a red square in the figure), we then use the lineage tree ${\mathcal {T}}_{t_{i+1}}$ to approximate the distribution ${\hat{p}}^s_{t_i}$ that would have been obtained at $t_i$ with the drift $-\nabla \left( \Psi + \Psi ^{t_{i+1}}\right)$. Note that without bias, that is with $\Psi ^{t_i}=cste$ for all *i*, each pair $({\hat{p}}_{t_i}, {\hat{p}}_{t_{i+1}})$ would be directly compatible and this scheme would consist only in a column instead of an array.

The principles of the method are illustrated in Fig. 6, and fuller details on each step can be found in Appendix 8.

The second step simply consists in reconstructing, from the pair of collection of marginal distributions ($\{\rho _{t_i}^s(\tilde{m})\}_{\tilde{m} \in {\mathbb {N}}}, \{\rho _{t_{i+1}}$), the pair of reweighted marginal distributions $(p_{t_i}^s, p_{t_{i+1}})$. From Corollary 11, it is thus compatible for the drift $-\nabla \left( \Psi + \Psi ^{t_{i+1}}\right)$.

**Fig. 7:**
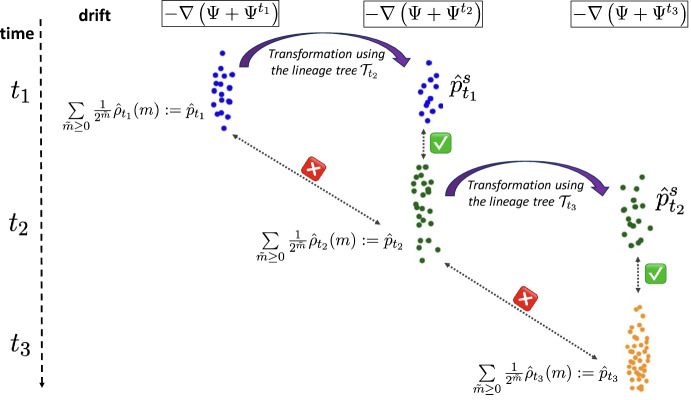
Estimation of the method for controlling the bias described in Section 5. Simulations of the second branching SDE described in Appendix 7 for (A) without subsampling, (B)-(C) subsampling with a decreasing rate from 1 to 0.05. In (C), the method described in Section 8.1 is applied to reconstruct pairs of timepoints that are compatible with the SDE (27). (E)-(F)-G reconstruction with the MFL method of the trajectories of the SDE from the datasets of (A)-(B)-(C) respectively. We also represent for each cell the velocity field estimated with the method described in Section 4.2 (in black), compared to the real one (in red). In (D) and (H), we compare the cumulated RMS distance between the reconstructed empirical distributions at each timepoint and the one obtained by simulating cells from the empirical distribution reconstructed at the previous timepoint, for the datasets of (A)-(B)-(C) and (D)-(E)-(F) respectively.

Figure 7 presents the results of applying this bias control method to data obtained from the same potential as Fig. 5. For each timepoint $t_i$, we compute the RMS distance between: i) the distribution obtained by simulating the SDE from the distribution at this timepoint (denoted $p_{t_i}^*$ for the standard MFL method, and $\mu _{t_i}^*$ for the algorithm presented in this section), and ii) the distribution computed by the method at the following timepoint (denoted $p_{t_{i+1}}^*$ for the standard MFL method, and $\nu _{t_i}^*$ for the algorithm presented in this section). The result provided is the cumulated RMS distance on the timepoints. As expected, our method reduces the bias between each pair of timepoints. In particular, for the reweighted marginal distributions (before applying the MFL algorithm), our method achieves a bias comparable with the case without subsampling. For the distributions reconstructed with the MFL algorithm, our method still allows for a bias reduction. In that case, the difference between the case with and without subsampling is less important since the MFL algorithm itself, by smoothing the errors, already provides good results. This is in line with the simulations in Fig. 5 which suggested that the bias induced by subsampling is small compared to the improvement reweighting provides.

#### Remark 13

The method presented in this section, in addition to losing the theoretical guarantees of the reweighting method, does not entirely remove the expected bias in the reconstructed drift. The results after correction in Fig. 7.F are not quite as good as the case without subsampling. The correction nevertheless has two advantages. First, the bias is better understood: each pair of distributions becomes compatible with the drift $-\nabla \left( \Psi + \Psi ^{t_i}\right)$. Moreover, it is expected to become very small when the timepoints are close: indeed, $-\nabla \Psi ^{t_i}$ should vanish when $t_i \rightarrow t_{i-1}$ as $S^{t_i}$ would be constant under Assumption 3 and uniform subsampling. We claim that this remaining bias reflects nonidentifiability in the data itself. Indeed, lineage tracing data are by nature unable to distinguish the cases in Figs. 1-B and 1-C, and thus reconstructing the characteristics of an SDE without any bias appears out of reach without new experimental techniques.

### Conditions for small bias

In this section, we provide some quantitative and qualitative analysis aimed at estimating, from some minimal knowledge on the branching rates, necessary conditions on the subsampling probability and the death rate for the bias from death and subsampling to be small.

On one side, an immediate consequence of Proposition 9 is that, when the extinction event is unlikely (*i.e* when Assumption ?? is satisfied), the marginal on ${\mathcal {X}}$ of the time-varying collection of distributions associated to the branching SDE with observable generation numbers and subsampling, ${\tilde{\rho }}_t:= \sum _{{\tilde{m}}} \rho _t({\tilde{m}})$, corresponds at every timepoint $t_i$ to the weak solution of the master equation on $\rho ^{t_i}$:

$$\begin{aligned} \partial _t \rho ^{t_i} = {\mathcal {L}}^{*}_{\Psi + \tau \ln S^{t_i}, \tau }\rho ^{t_i} + bS^{t_i} \rho ^{t_i}, \end{aligned}$$

with the initial condition $\rho ^{t_i}_0 = \mu _0$. If $S^{t_i}$ was constant, we would not have bias: thus ${\tilde{\rho }}$ would correspond to the solution of the the master equation describing the branching-free SDE Eq. (9) with an additional birth term. As birth can only add mass, no bias thus implies that for all *t*, *x*, ${\tilde{\rho }}_t(x) \ge p_t(x)$, where we recall that $(p_t)$ denotes the time-varying probabilistic distribution defined by (9) with the same initial condition $\mu _0$. By substituting Eq. (6) into this inequality, we thus obtain a necessary condition on the subsampling rate:

28 $$\begin{aligned} \forall t \in [0,T],\, \forall x \in {\mathcal {X}}:\, q(t) {\tilde{\rho }}_t(x) \ge p_t(x), \end{aligned}$$

$(\rho _t)$ denoting the time-varying marginal distribution on ${\mathcal {X}}$ of the same branching SDE without subsampling.

We can now combine Eq. (28) with Corollary 11 to determine the subsampling probability *q*(*t*) required for the reweighting method presented in Section 4.2.1 to be accurate in the limit of small noise, even with non-negligible branch survival probability $S^{t_i}$ (Eq (23)). Indeed, the drift bias has been explicitly identified and is equal to $-\tau \nabla \ln S^{t_i}$. Then, if the branching rates are smooth enough and the system generated by the gradient drift $-\nabla \Psi$ tends to not push the cells too far apart, we can reasonably expect the quantity $\nabla \log (S^{t_i})$ to remain $o(1/\tau )$ when $\tau$ is small. Thus, as $\tau \rightarrow 0$, for every timepoint $t_i$ the distribution $\bar{\rho }_{t_i}$ should converge to the temporal marginal distribution at $t_i$ of the ground-truth time-varying probabilistic distribution $(p_t)$ defined by (9). At the limit, the limiting factor is then the value of $\varepsilon _{t_i}$. Thus, as on every trajectory solution of the dynamical system ${\dot{\phi }} = -\nabla \Psi (t, \phi )$, we must have $\rho _t(x) = e^{\int _0^t (b-d)(s, \phi (s))\mathrm ds}p(t)$, Eq. (28) provides a necessary condition for the bias to be small:

29 $$\begin{aligned} q(t)e^{\int _0^t (b-d)(s, \phi (s))\mathrm ds} \ge 1 \end{aligned}$$

for all $t \in [0,T]$. This last inequality can be interpreted as a way of using prior knowledge on the birth and death rates to estimate the minimum subsampling probability required for accurate results from the reweighting method. For example, taking $d = 0$, a rough condition on *q* becomes:

$$\begin{aligned} q(t) \ge e^{- t\min \limits _{[0,t]\times {\mathcal {X}}} b}. \end{aligned}$$

Similarly, if $d \ne 0$ and $q = 1$ uniformly on [0, *T*], a rough condition for the relation (29) to be satisfied is that $b - d \ge 0$ on $[0,T] \times {\mathcal {X}}$.

We expect this analysis in the limit of small noise to remain relevant in the realistic situation where the diffusion coefficient $\tau$ is not close to 0. Indeed, if the relation (28) is satisfied, the probability of a cell to have a descendant subsampled at each timepoint ought to be high everywhere, and then the bias defined by $\tau \nabla \ln S^{t_i}$ is close to 0. Eq. (28), therefore, is both a necessary condition for the reweighting method to be accurate when $\tau \rightarrow 0$ and a heuristic quantitative condition for the bias to be reasonably small for any $\tau$.

## Discussion

We have shown in this article that observing the generation numbers associated to the leaves of a tree characterizing a realization of a branching SDE is sufficient, in certain conditions, to deconvolve proliferation from the dynamics of individual cells. Our work extends the mathematical theory of trajectory inference developed for SDEs without proliferation to the branching case with similar theoretical guarantees and computational cost. Using the generation numbers, we are able to not only accurately estimate the drift of the SDE, as has been done for the case without branching, but also learn the proliferation rate with no prior knowledge. In particular, in the limit of infinitely close timepoints, we can rigorously reconstruct the law of the SDE modeling the motion of cells in gene expression space from time-series of measures from the process with proliferation, if we have access to the *observable generation numbers* and we observe all the leaves of at least one full tree per timepoint and there is no death (Theorem 7).

These results demonstrate that much of the information in the lineage trees is not needed for reconstructing the trajectories of branching processes when there is no death nor subsampling. Our algorithm of Section 4.2.2 does not use the times of last common ancestors between any pair of cells and the joint distribution of leaves. If cells are subsampled and can die, we have shown that this method generates a bias in the reconstructed drift and branching rate. This bias is expected to be small in biologically relevant scenarios where the probability of having at least one observed descendant does not vary dramatically across cell. Section 5.3 provides a heuristic analysis linking the subsampling rate to the branching rates required for this assumption. This bias can also be partially removed using additional information from the lineage tree via the algorithm of Section 5.2, albeit without the theoretical guarantees proved for the two cases above.

The main property underlying our results, that the reweighted marginal distribution on ${\mathcal {X}}$ of a joint distribution on ${\mathbb {N}} \times X$ of a branching SDE corresponds to the marginal probabilistic distribution of the SDE without branching (see Corollary 6), with a possible bias due to death (see Corollary 11), holds for general branching processes. Although we focused in this article on branching SDEs, these properties could be directly applied to account for branching in models with other experimentally-relevant stochastic processes, such as the gene regulatory network inference method based on switching ODEs we recently introduced (Ventre et al. 2023).

Altogether, we believe that these results present a major theoretical advance in the nascent field of single-cell data analysis with lineage tracing. We go beyond the point of view developed in methods previously published to analyze these data (Forrow and Schiebinger 2021; Wang et al. 2022; Lange et al. 2023), which were only interested in reconstructing couplings between empirical measures of cells. As the notion of coupling between cells of empirical non-probabilistic measures is itself hard to define properly, it seems difficult to relate the results provided by these methods to the notion of path-measure of an underlying branching process, which is the aim of trajectory inference. To tackle this problem, our method reconstructs well defined couplings between cells belonging to the temporal marginal probabilistic distributions of the SDE underlying the observed branching SDE: considering these probabilistic distributions instead of the non-probabilistic observable measures is one of the major strengths of our work. Moreover, we emphasize that this underlying SDE is the main mathematical object of interest for biologists, as it shapes the called Waddington landscape (Waddington 2014; Huang et al. 2005) of differentiation, which encodes the regulatory mechanisms controlling cell fates. In reconstructing the law of this SDE from scRNA-seq measurements with lineage tracing, our method extracts the most important information from the data. Combined with estimation of the branching rate, we achieve a full understanding of the branching SDE when there is no death.

## Parameters of the simulations

The applications of this article focus on two very simple potentials defined in ${\mathbb {R}}^3$, which are both associated to non-constant birth rates. For both potentials, the branching rate is built such that it is constant in every potential well: this is in line with the interpretation of the potential wells as cell types, and the fact to consider that the stochasticity is characterized by the transitions between these cell types (Ventre et al. 2021).

We define $A = (1.5, 0, 0), B = (-1.5, 0, 0)$. The first potential, corresponding to the simulations of the first row of Fig. 2 and of the simulations of Figures 3 and 4 is the following:

$$\begin{aligned} \forall x = (x_0, x_1, x_2) \in {\mathbb {R}}^3:\,V_1(x,t) = (x_0 - A)^2 (x_0 - B)^2 + 10 (x_1 + t)^2 + 10 (x_2)^2. \end{aligned}$$

The birth rate is:

$$\begin{aligned} b_1(x, t) = 3(\tanh (2x_0) + 1). \end{aligned}$$

It has two wells at any timepoint.

The second one, corresponding to the simulations of the second row of Fig. 2 and of the simulations of Figures 5 and 7 is the following:

$$\begin{aligned} \forall x = (x_0, x_1, x_2) \in {\mathbb {R}}^3:\,V_2(x,t) = 10*(\cos (x_0) - \text {abs}(x_0)) + 10 (x_1 + t)^2 + 10 (x_2)^2. \end{aligned}$$

The birth rate is:

$$\begin{aligned} b_2(x, t) = {\left\{ \begin{array}{ll} 2(\tanh (x_0/5) + 1) & \text { if } x_1 \le -0.5 \text { and } x_0 \le 6,\\ 4(\tanh (x_0/5) + 1) & \text { if } x_1 \le -0.5 \text { and } x_0> 6,\\ 10 & \text { if } x_1 > -0.5. \end{array}\right. } \end{aligned}$$

It has an infinite number of wells, but at the time of simulations the SDE has only explored between 4 and 6 wells at the last timepoint (see Fig. 2).

For the sake of simplicity, we consider the death rates $d_1 = d_2 = 0$ on ${\mathbb {R}}^3$.

## Algorithm for the heuristic method described in Section 5

We present here the details of the algorithm presented in Section 5.2 aiming to use the lineage information to reduce the bias arising in the reweighting method in case of death and/or subsampling. The results obtained with this method are illustrated in Fig. 7.

The principle of this heuristic method relies on an additional assumption: we consider that for every cell subsampled at a timepoint $t_{i+1}$, an ancestor of it would have been subsampled at the previous timepoint $t_i$ with high probability. The underlying idea is that the total mass of the process is expected to increase in [0, *T*] and that the subsampling probability *q*(*t*) is decreasing (*i.e* there are more and more cells in the organism but we subsample a smaller and smaller fraction of them). Thus, if the distribution of an area $A_{i+1} \subset {\mathcal {X}}$ is high enough at time $t_{i+1}$ for a cell to be subsampled, the ancestors of the cells in this area should be mostly concentrated at time $t_i$ in an area $A_i$, such that the probability of subsampling a cell there should be high too.

Note that if the subsampling probability is uniformly 1 in [0, *T*], so that the condition for a cell to be subsampled is only that it is alive, this hypothesis only means that every cell alive at a certain time has an ancestor alive at earlier timepoints, which is obviously verified.

### Reconstruction of the collection of distributions $\{\rho _{t_i}^s(m)\}_{m \in {\mathbb {N}}}$

In practice, we only have access at each timepoint $t_i$ to a collection of empirical (non probabilistic) distributions $\{{\hat{\rho }}^s_{t_i}({\tilde{m}})\}_{{\tilde{m}} \in {\mathbb {N}}}$, each of them corresponding to a sum of diracs on ${\mathcal {X}}$ reweighted by the number of trees $K_i$ observed at $t_i$. From each pair of collection of empirical distributions $(\{{\hat{\rho }}_{t_i}({\tilde{m}})\}_{{\tilde{m}} \in {\mathbb {N}}}, \{{\hat{\rho }}_{t_{i+1}}({\tilde{m}})\}_{{\tilde{m}} \in {\mathbb {N}}})$, we propose thus to estimate the collection of distributions $\{{\hat{\rho }}_{t_i}^s({\tilde{m}})\}_{{\tilde{m}} \in {\mathbb {N}}}$ using the two-steps algorithm presented in Section 5.2. More precisely, step 2 consists in computing a transport map $T^* \in {\mathcal {P}}({\mathcal {X}} \times {\mathcal {X}})$:

$$\begin{aligned} T^* = \mathop {\mathrm {arg\,min}}\limits \limits _{T} \sum \limits _{{\tilde{m}} \in {\mathbb {N}}, {\tilde{m}}' \in {\mathbb {N}}}\sum \limits _{({\tilde{m}},x) \in {\hat{\rho }}_{t_i}({\tilde{m}}), ({\tilde{m}}',y) \in {\hat{\rho }}_{t_i}^s({\tilde{m}})}\left( ||x - y ||^2 + |\sqrt{{\tilde{m}}} - \sqrt{{\tilde{m}}'}|\right) T(({\tilde{m}}', \mathrm dy), ({\tilde{m}}, x)), \end{aligned}$$

such that:

$$\sum \limits _{{\tilde{m}} \in {\mathbb {N}}}\sum \limits _{({\tilde{m}},x) \in {\hat{\rho }}_{t_i}({\tilde{m}})}T^*(({\tilde{m}}', \mathrm dy), ({\tilde{m}}, x)) = \rho _{t_i}^s({\tilde{m}}', y)$$, and

$$\sum \limits _{{\tilde{m}}' \in {\mathbb {N}}}\sum \limits _{({\tilde{m}}',y) \in {\hat{\rho }}_{t_i}^s({\tilde{m}})}T(({\tilde{m}}', \mathrm dy), ({\tilde{m}}, x)) = \rho _{t_i}({\tilde{m}}, x)$$.

We can thus improve the estimate of the collection of marginal distributions $\{{\hat{\rho }}_{t_i}^s(\tilde{m})\}_{\tilde{m} \in {\mathbb {N}}}$ using this transport map. For every cell $({\tilde{m}},x)$ belonging to the support of ${\hat{\rho }}^{s}_{t_i}({\tilde{m}})$, substitute *x* by $x'$ defined as:

$$\begin{aligned} x' = \sum \limits _{{\tilde{m}}' \in {\mathbb {N}}} \sum \limits _{({\tilde{m}}',y) \in {\hat{\rho }}_{t_i}({\tilde{m}})} y T(({\tilde{m}}', \mathrm dy), ({\tilde{m}}, x)). \end{aligned}$$

$\{{\hat{\rho }}_{t_i}^s(\tilde{m})\}_{\tilde{m} \in {\mathbb {N}}}$ are then defined as the empirical measure defined on these new positions $x'$ together with the same generation numbers.

### Trajectory inference for a SDE with a known drift with reduced bias

With this new sequence of collection of empirical distributions $(\{{\hat{\rho }}^s_{t_i}({\tilde{m}})\}_{{\tilde{m}} \in {\mathbb {N}}})_{i=1,\cdots ,T}$ in hands, we can then build a minimization problem allowing to regularize across the timepoints, as in Section 4.2. For this, we aim to extend the MFL algorithm used in Section 4.2 for 2 sequences of empirical distributions: the observed sequence of reweighted distributions $({\hat{p}}_{t_i})_{i=1,\cdots ,T}$ and the reconstructed sequence of reweighted marginal distributions $({\hat{p}}^s_{t_i})_{i=1,\cdots ,T-1}$.

In addition to minimizing a Schrödinger problem between each pair of reweighted marginal distributions ($\hat{p}^s_{t_i}$, $\hat{p}_{t_{i+1}}$), to chain all these pairs together by minimizing a Wasserstein distance between  and $\hat{p}^s_{t_i}$, as by construction $\hat{p}^s_{t_i}$ is expected to be close to $\hat{p}_{t_i}$ when $t_i$ and $t_{i+1}$ are close.

We thus find two new sequences of empirical distributions $\mu _{t_1},\cdots ,\mu _{t_N}$ and $\nu _{t_1},\cdots ,\nu _{t_N}$, each being described by an empirical measure with *M* diracs, solving the following problem:

30 $$\begin{aligned} \inf \limits _{\mu , \nu }\left\{ G(\mu ,\nu ) + \tau F(\mu ,\nu ) \right\} , \end{aligned}$$

with

$$\begin{aligned} G(\mu ,\nu )&= \sum \limits _{i = 1}^{N-1} T_{\tau _i}(\mu _{t_i}, \nu _{t_{i}}) + \sum \limits _{i = 1}^{N-1} OT(\nu _{t_{i}}, \mu _{t_{i + 1}}) \\&+ \frac{1}{\lambda }\sum _{i=1}^{N - 1} \Delta t_i\left[ H\left( {\hat{\rho }}_{t_i + 1} | \Phi _h * \nu _{t_i}\right) + H\left( {\hat{\rho }}^s_{t_i} | \Phi _h * \mu _{t_i}\right) \right] , \end{aligned}$$

and $$F(\mu ,\nu ) = \sum \limits _{i = 1}^{N-1} H(\mu _{t_i}) + H(\nu _{t_{i}})$$.

As in Zhang et al. (2022), the functional to minimize is jointly convex in the $\mu _{t_i}$ and $\nu _{t_i}$ as a sum of supremum of linear forms and convex functions, and we can use a similar algorithm to solve the minimization problem and find the optimal empirical probabilistic distributions corresponding to the optimal pairs $(\mu _{t_i}^*, \nu _{t_i}^*)$. The code of these algorithms is available on Github.

## Footnotes

1 Note that the function *h* is generally introduced without the $1-x$ because the expected value of $1 - \frac{\mathrm dR}{\mathrm dW^\tau }$ is always 0 when we consider path-measures whose temporal marginals integrate to 1, which is not always the case in this article.

## Declarations

### Code availability

The code for reproducing the figures of this article is available at https://github.com/eliasventre/LTreweighting.
